# Caspase-11 Activation in Response to Bacterial Secretion Systems that Access the Host Cytosol

**DOI:** 10.1371/journal.ppat.1003400

**Published:** 2013-06-06

**Authors:** Cierra N. Casson, Alan M. Copenhaver, Erin E. Zwack, Hieu T. Nguyen, Till Strowig, Bahar Javdan, William P. Bradley, Thomas C. Fung, Richard A. Flavell, Igor E. Brodsky, Sunny Shin

**Affiliations:** 1 Department of Microbiology, Perelman School of Medicine, University of Pennsylvania, Philadelphia, Pennsylvania, United States of America; 2 Department of Pathobiology, School of Veterinary Medicine, University of Pennsylvania, Philadelphia, Pennsylvania, United States of America; 3 Department of Immunobiology and Howard Hughes Medical Institute, Yale University School of Medicine, New Haven, Connecticut, United States of America; Tufts University School of Medicine, United States of America

## Abstract

Inflammasome activation is important for antimicrobial defense because it induces cell death and regulates the secretion of IL-1 family cytokines, which play a critical role in inflammatory responses. The inflammasome activates caspase-1 to process and secrete IL-1β. However, the mechanisms governing IL-1α release are less clear. Recently, a non-canonical inflammasome was described that activates caspase-11 and mediates pyroptosis and release of IL-1α and IL-1β. Caspase-11 activation in response to Gram-negative bacteria requires Toll-like receptor 4 (TLR4) and TIR-domain-containing adaptor-inducing interferon-β (TRIF)-dependent interferon production. Whether additional bacterial signals trigger caspase-11 activation is unknown. Many bacterial pathogens use specialized secretion systems to translocate effector proteins into the cytosol of host cells. These secretion systems can also deliver flagellin into the cytosol, which triggers caspase-1 activation and pyroptosis. However, even in the absence of flagellin, these secretion systems induce inflammasome activation and the release of IL-1α and IL-1β, but the inflammasome pathways that mediate this response are unclear. We observe rapid IL-1α and IL-1β release and cell death in response to the type IV or type III secretion systems of *Legionella pneumophila* and *Yersinia pseudotuberculosis*. Unlike IL-1β, IL-1α secretion does not require caspase-1. Instead, caspase-11 activation is required for both IL-1α secretion and cell death in response to the activity of these secretion systems. Interestingly, whereas caspase-11 promotes IL-1β release in response to the type IV secretion system through the NLRP3/ASC inflammasome, caspase-11-dependent release of IL-1α is independent of both the NAIP5/NLRC4 and NLRP3/ASC inflammasomes as well as TRIF and type I interferon signaling. Furthermore, we find both overlapping and non-redundant roles for IL-1α and IL-1β in mediating neutrophil recruitment and bacterial clearance in response to pulmonary infection by *L. pneumophila*. Our findings demonstrate that virulent, but not avirulent, bacteria trigger a rapid caspase-11-dependent innate immune response important for host defense.

## Author Summary

The inflammasome, a multiprotein complex, is critical for host defense against bacterial infection. The inflammasome activates the host protease caspase-1 to process and secrete IL-1β. Another caspase, caspase-11, can cause cell death and IL-1α release. The bacterial signals that trigger caspase-11 activation are poorly understood. A common feature of many bacterial pathogens is the ability to inject virulence factors and other bacterial molecules into the host cell cytosol by means of a variety of virulence-associated secretion systems. These secretion systems can introduce bacterial flagellin into the host cytosol, which leads to caspase-1 activation and cell death. However, many bacteria lack or down-regulate flagellin yet still activate the inflammasome. Here, we show that the type IV secretion system of *Legionella pneumophila* and the type III secretion system of *Yersinia pseudotuberculosis* rapidly trigger caspase-11 activation in a flagellin-independent manner. Caspase-11 activation mediates two separate inflammasome pathways: one leading to IL-1β processing and secretion, and one leading to cell death and IL-1α release. Furthermore, we find these caspase-11-regulated cytokines are critical for neutrophil recruitment to the site of infection and clearance of non-flagellated *Legionella in vivo*. Overall, our findings show that virulent bacteria activate a rapid caspase-11-dependent immune response that plays a critical role in host defense.

## Introduction

Antibacterial defense is initiated by germline-encoded pattern recognition receptors (PRRs), which detect conserved pathogen-associated molecular patterns (PAMPs) [1]–[3]. Plasma membrane-bound PRRs, such as the Toll-like receptors (TLRs), detect PAMPs present in the extracellular space and endosomal compartments, whereas cytosolic PRRs, such as the NOD-like receptors (NLRs), survey the host cytosol for the presence of invasive pathogens [3]–[7]. Invasive microorganisms or other cellular stresses induce assembly of cytosolic protein complexes known as inflammasomes, which play a critical role in host defense [8]–[11]. Inflammasomes respond to a wide variety of activators, including bacterial pore-forming toxins and bacterial PAMPS, such as flagellin or RNA [12]–[18]. Particular NLRs respond to their cognate stimuli and recruit the adapter protein ASC and pro-caspase-1 through homotypic protein-protein interactions between pyrin domains and caspase recruitment domains (CARD), leading to autoprocessing and activation of caspase-1 [19]–[23]. Caspase-1 is responsible for processing and secreting IL-1 family cytokines and mediates a proinflammatory cell death termed pyroptosis [9], [11], [24], [25].

Caspase-11 participates in the activation of a non-canonical inflammasome that induces cell death and the secretion of IL-1α and IL-1β in response to Gram-negative pathogens, such as *Escherichia coli* and *Vibrio cholerae*, and to particular toxins, such as the cholera toxin B subunit [26]–[29]. This non-canonical, caspase-11-dependent response to Gram-negative bacteria is independent of virulence-associated secretion systems that deliver bacterial molecules into the host cytosol and requires LPS-induced TLR4 signaling through the adaptor TIR-domain-containing adaptor-inducing interferon-β (TRIF) and TRIF-dependent type I interferon (IFN) production. Type I IFN signaling through the type I IFN receptor (IFNAR) is required for caspase-11 upregulation and activation, but how type I IFN mediates activation of caspase-11 is not well-defined [27]–[29]. Caspase-11 contributes to NLRP3-dependent activation of caspase-1 and subsequent caspase-1-dependent IL-1β secretion and cell death. Caspase-11 also facilitates an NLRP3- and caspase-1-independent pathway that results in cell death and release of IL-1α [26]–[29]. This caspase-11-dependent, caspase-1-independent pathway is responsible for LPS-induced septic shock *in vivo* [26], [30]. Although caspase-11 is activated in response to signals from Gram-negative pathogens and certain pore-forming toxins, whether caspase-11 contributes to inflammasome activation in response to virulence-associated secretion systems that deliver bacterial ligands into host cytosol is unknown.

Bacterial pathogens use evolutionarily conserved secretion systems, such as type III or type IV secretion systems (T3SS or T4SS), to translocate effector proteins into the cytosol of host cells [31], [32]. In addition to bona fide virulence factors, these secretion systems also translocate bacterial molecules such as flagellin or structural components of the secretion machinery itself, which results in inflammasome activation [14], [16], [33]–[36]. *Legionella pneumophila*, an opportunistic pathogen that causes a severe pneumonia known as Legionnaires' disease [37], [38], utilizes its *dot/icm*-encoded T4SS as a virulence factor to translocate bacterial effector proteins into the host cell cytosol and establish a replicative vacuole [39]–[46]. *L. pneumophila* induces T4SS-dependent inflammasome activation through two genetically distinct pathways [47]. T4SS-mediated translocation of flagellin into the cytosol triggers caspase-1 activation and pyroptosis through the NLR NAIP5 in conjunction with another NLR, NLRC4 [16], [36], [47]–[50]. Caspase-1 activation is also triggered independently of the NLRC4/flagellin pathway through the adaptor protein ASC, but the bacterial factor that is recognized and the upstream proteins that regulate this pathway remain unknown [47], [51]. However, although ASC is necessary for robust secretion of IL-1β in response to *L. pneumophila* as well as a number of pathogens, such as *Salmonella* or *Yersinia* species which employ T3SSs, ASC is dispensable for induction of pyroptosis that is rapidly triggered in response to these infections. We therefore considered the possibility that in addition to its role in delayed inflammasome activation in response to Gram-negative bacteria, caspase-11 might participate in rapid cell death and release of IL-1α in response to the presence of bacterial pathogens that access the host cell cytosol by means of type IV and type III secretion systems.

Here, we demonstrate that IL-1α and IL-1β are rapidly released in response to bacterial T4SS activity independently of bacterial flagellin. In this system, we find IL-1β secretion requires caspase-1, but caspase-1 is dispensable for cell death and IL-1α release in response to a functional *L. pneumophila* T4SS. Instead, caspase-11 is required for both IL-1α release and cell death in response to *L. pneumophila* T4SS activity. Consistent with recent findings, caspase-11 contributes to optimal NLRP3-mediated caspase-1 activation and IL-1β secretion in response to *L. pneumophila*. However, caspase-11-dependent IL-1α release and cell death in *L. pneumophila*-infected cells are independent of the NAIP5/NLRC4 and NLRP3/ASC inflammasomes. In contrast to the role of TRIF and IFNAR in the response against Gram-negative bacteria, caspase-11 activation and cytokine release in response to the T4SS of *L. pneumophila* are independent of both TRIF and IFNAR signaling. We further demonstrate that T3SS activity of the unrelated pathogen *Yersinia pseudotuberculosis* induces a similarly rapid caspase-11-dependent response that also leads to cell death and release of IL-1α and IL-1β. Finally, we find that both IL-1α and IL-1β are critical *in vivo* for neutrophil recruitment and bacterial clearance. Overall, our data show that caspase-11 is poised to respond robustly to a conserved feature of pathogenic bacteria, bacterial access to the host cytosol through specialized secretion systems. This establishes caspase-11 as a critical regulator of immune system-mediated discrimination of pathogenic and nonpathogenic bacteria.

## Results

### LPS priming induces rapid IL-1α and IL-1β secretion in response to *L. pneumophila* T4SS activity

**Figure 1:**
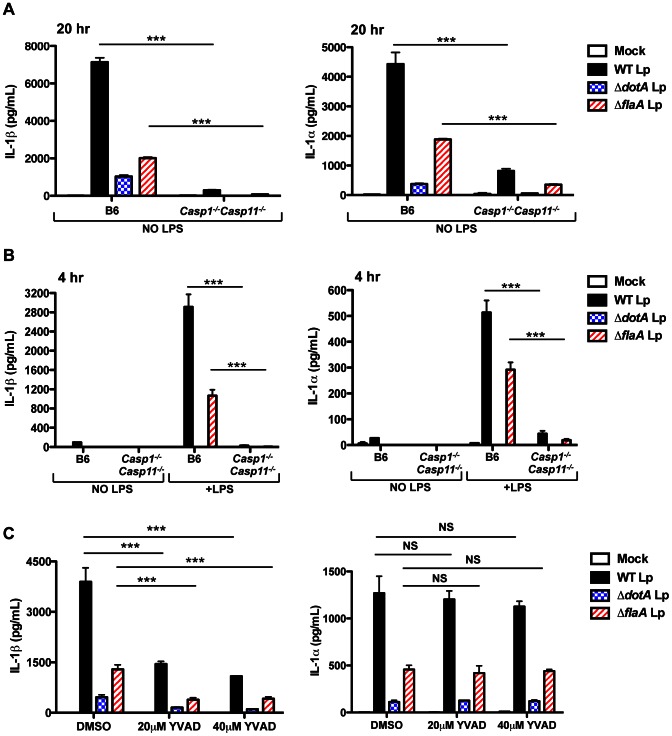
LPS priming induces rapid IL-1α and IL-1β secretion in response to *L.* *pneumophila* T4SS activity. (**A**) Unprimed B6 or *Casp1^−/−^Casp11^−/−^* BMDMs were infected with WT *L. pneumophila* (WT Lp), Δ*dot* Lp, Δ*flaA* Lp, or PBS (mock infection) for 20 hours. (**B**) B6 or *Casp1^−/−^Casp11^−/−^* BMDMs were either unprimed or primed with 0.5 µg/mL LPS for 2.5 hours and infected with WT Lp, Δ*dotA* Lp, Δ*flaA* Lp, or PBS for 4 hours. (**C**) B6 BMDMs were pretreated with either 20 µM or 40 µM of the caspase-1 inhibitor YVAD-cmk or DMSO vehicle control for 0.5 hours and infected with WT Lp, Δ*dotA* Lp, Δ*flaA* Lp, or PBS for 20 hours. Levels of IL-1α and IL-1β in the supernatants were measured by ELISA. Graphs show the mean ± SEM of triplicate wells. Data are representative of three or four independent experiments. *** is p<0.001 by 2-way ANOVA with Bonferroni post-test. NS is not significant.

*L. pneumophila* infection induces IL-1α and IL-1β secretion that requires T4SS activity [47], [52]. IL-1β secretion is regulated by a flagellin-dependent NAIP5/NLRC4 inflammasome and a poorly defined ASC inflammasome that both activate caspase-1 [47], [51]. The mechanisms underlying IL-1α secretion are less clear, but IL-1α secretion is still robustly induced by flagellin-deficient *L. pneumophila*, which do not activate the NAIP5/NLRC4 inflammasome [52]. Recent studies have described a non-canonical inflammasome triggered in response to Gram-negative bacteria. This non-canonical inflammasome requires lipopolysaccharide (LPS) for the upregulation and activation of caspase-11 and subsequent IL-1α and IL-1β release [26]–[29]. Whether caspase-11 is also activated in response to bacteria that use specialized secretion systems to translocate bacterial molecules into the host cytosol is unknown. We thus hypothesized that LPS priming would upregulate caspase-11, pro-IL-1α, and pro-IL-1β and allow for more robust and rapid IL-1α and IL-1β secretion in response to T4SS activity. To test this, we first compared IL-1α and IL-1β release in unprimed and LPS-primed bone marrow-derived macrophages (BMDMs). As shown previously [48], [52], unprimed BMDMs secrete robust levels of IL-1α and IL-1β by 20 hours post-infection with wild-type *L. pneumophila* (WT Lp) (Figure 1A). Slightly attenuated levels of secreted IL-1α and IL-1β are observed with flagellin-deficient *L. pneumophila* (Δ*flaA* Lp), which do not activate the NAIP5/NLRC4 inflammasome [17], [18]. Secretion of both cytokines is significantly diminished during infection with *L. pneumophila* lacking DotA, an essential component of the T4SS (Δ*dotA* Lp), and is significantly diminished in caspase-1/caspase-11-deficient (*Casp1^−/−^Casp11^−/−^*) macrophages as well (Figure 1A). The diminished IL-1 secretion induced by Δ*dotA* Lp is not due to a lack of pro-IL-1 production, as Δ*dotA* Lp and WT Lp induce robust levels of pro-IL-1β (Figure S1A). At 4 hours post-infection, unprimed macrophages do not secrete IL-1 (Figure 1B). However, LPS-primed cells rapidly secrete IL-1α and IL-1β, and this secretion is abrogated in *Casp1^−/−^Casp11^−/−^* macrophages (Figure 1B). Secretion of IL-18, another IL-1 family cytokine, also requires T4SS activity and is eliminated in *Casp1^−/−^Casp11^−/−^* cells (Figure S1B). Comparable levels of the caspase-1/caspase-11-independent cytokines IL-12 and TNF-α are secreted in the absence and presence of LPS priming (Figure S1C–D). These data suggest that LPS priming upregulates a factor required for rapid IL-1α and IL-1β release in response to *L. pneumophila* T4SS activity.

### Caspase-1 catalytic activity is required for IL-1β but not IL-1α secretion

Secretion of IL-1β in response to both canonical and non-canonical inflammasome activation requires caspase-1 [26], [53], [54]. In contrast, IL-1α release downstream of the non-canonical inflammasome depends on caspase-11, and does not require caspase-1 [26]. To test if the catalytic activity of caspase-1 is required for IL-1α secretion in response to *L. pneumophila*, we inhibited caspase-1 catalytic activity with the pharmacological inhibitor YVAD-cmk (YVAD). Consistent with previous studies [53], IL-1β secretion in response to *L. pneumophila* is substantially inhibited by YVAD. However, YVAD has no effect on IL-1α secretion, indicating that IL-1α release in response to *L. pneumophila* does not require caspase-1 catalytic activity (Figure 1C), as has been shown for other inflammasome activators [55]. Given that IL-1α secretion occurs more rapidly upon LPS priming, is abrogated in *Casp1^−/−^Casp11^−/−^* macrophages, and does not require caspase-1 catalytic activity, we considered the possibility that caspase-11 might participate in inflammasome activation during *L. pneumophila* infection.

### Caspase-11 contributes to inflammasome activation in response to flagellin-deficient *L. pneumophila*

**Figure 2:**
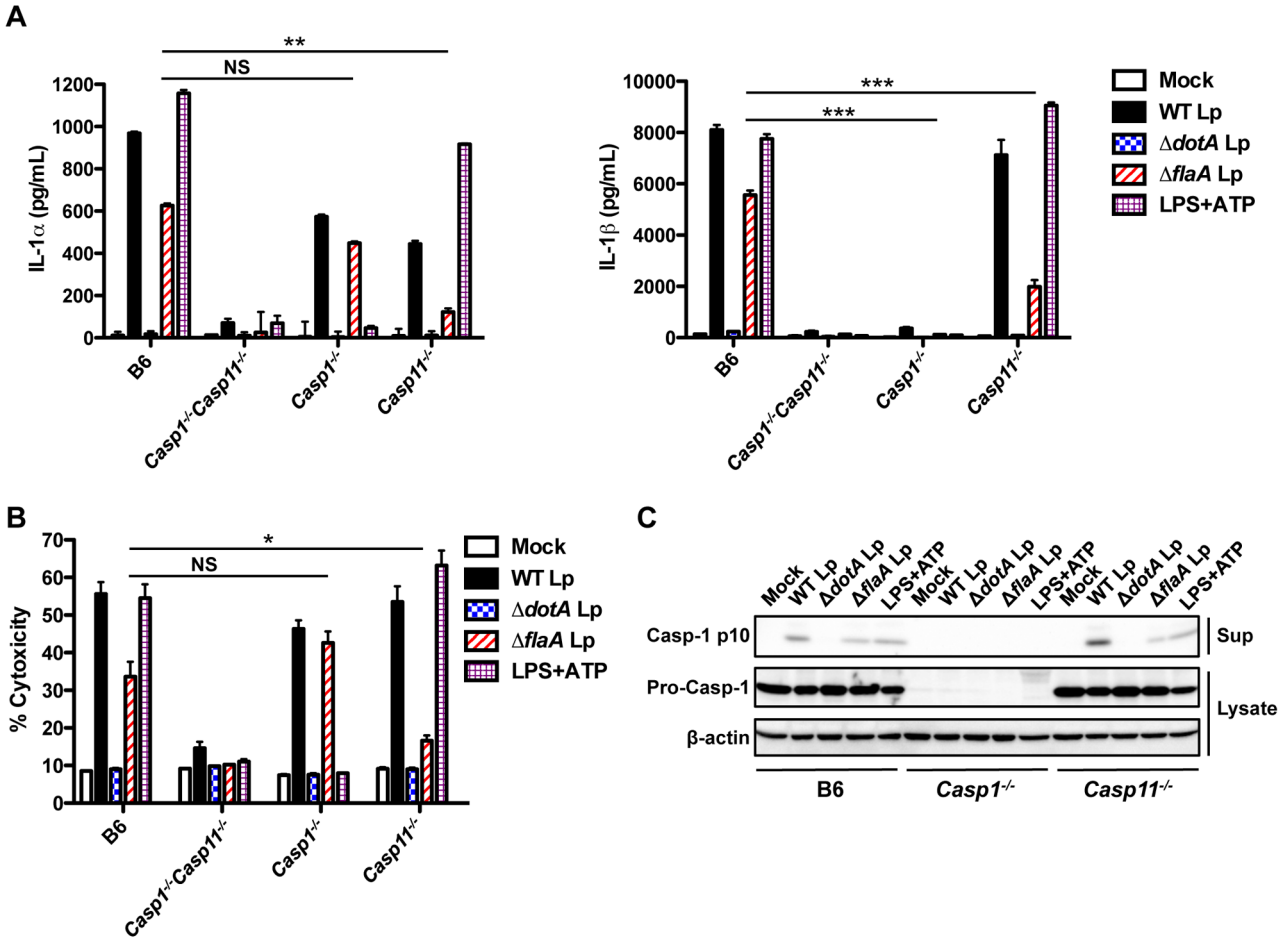
Caspase-11 controls the release of IL-1α and IL-1β and pyroptosis in response to flagellin-deficient *L.* *pneumophila*. B6, *Casp1^−/−^Casp11^−/−^*, *Casp1^−/−^*, or *Casp11^−/−^* BMDMs were primed with 0.5 µg/mL LPS for 2.5 hours and infected with WT *L. pneumophila* (WT Lp), Δ*dotA* Lp, Δ*flaA* Lp, or PBS (mock infection) or treated with 2.5 mm ATP for 1(C) or 4(A,B) hours. (**A**) Levels of IL-1α and IL-1β in the supernatants were measured by ELISA. Graphs show the mean ± SEM of triplicate wells. (**B**) Cell death (% cytotoxicity) was measured by LDH release into the supernatants relative to Triton X-100-lysed cells. Graphs show the mean ± SEM of triplicate wells. (**C**) Levels of processed caspase-1 (casp-1 p10) in the supernatants and full-length caspase-1 (pro-casp-1) and β-actin in the cell lysates were determined by immunoblot analysis. Data are representative of three independent experiments. *** is p<0.001 by two-way ANOVA with Bonferroni post-test, ** is p<0.01 by two-way ANOVA with Bonferroni post-test, and * is p<0.05 by unpaired t-test. NS is not significant.

To test the genetic requirement for caspase-11 in the inflammasome response to *L. pneumophila*, we infected BMDMs from either caspase-1-deficient (*Casp1^−/−^*) or caspase-11-deficient (*Casp11^−/−^*) mice. In the absence of flagellin, caspase-11 is required for IL-1α secretion, whereas it is not essential for IL-1β secretion but contributes to maximal secretion (Figure 2A). These data suggest that caspase-11 is activated in response to *L. pneumophila* infection independently of flagellin. Indeed, there is robust processing and secretion of caspase-11 in response to WT and Δ*flaA* Lp (Figure S2). In accordance with previous findings [26], [53], caspase-1 is absolutely required for IL-1β secretion. In contrast, we observe robust IL-1α release even in the absence of caspase-1. Both IL-1α and IL-1β release in response to Δ*flaA* Lp are caspase-11-dependent in both primed and unprimed macrophages (Figures 2, S3A–B), making *L. pneumophila* distinct from other Gram-negative bacteria that require priming to induce robust caspase-11 upregulation and activation [27]. Thus, while caspase-11 contributes to maximal caspase-1-dependent IL-1β secretion, it is both necessary and sufficient for IL-1α release in response to flagellin-deficient *L. pneumophila*.

Cell death in B6 BMDMs is partially flagellin-dependent, but is flagellin-independent in *Casp1^−/−^* BMDMs (Figure 2B). Importantly, cell death in response to flagellin-deficient *L. pneumophila* requires caspase-11, thus correlating caspase-11-dependent cell death with IL-1α release from host cells. In contrast, and consistent with previous findings [26], LPS+ATP induces canonical caspase-1-dependent pyroptosis and secretion of IL-1α and IL-1β that is independent of caspase-11. Because caspase-1 must be processed to mediate IL-1β secretion [53], we examined whether caspase-1 processing is decreased in the absence of caspase-11, which could account for the decreased IL-1β secretion in response to Δ*flaA* Lp. Caspase-1 processing is slightly attenuated but not abrogated in response to Δ*flaA* Lp in *Casp11^−/−^* macrophages, consistent with the slight decrease in IL-1β secretion (Figures 2C, S3C). Thus, flagellin-deficient *L. pneumophila* trigger a canonical caspase-1-dependent inflammasome as well as a non-canonical caspase-11-dependent inflammasome.

### Caspase-11 activation is independent of ASC and NLRC4

**Figure 3:**
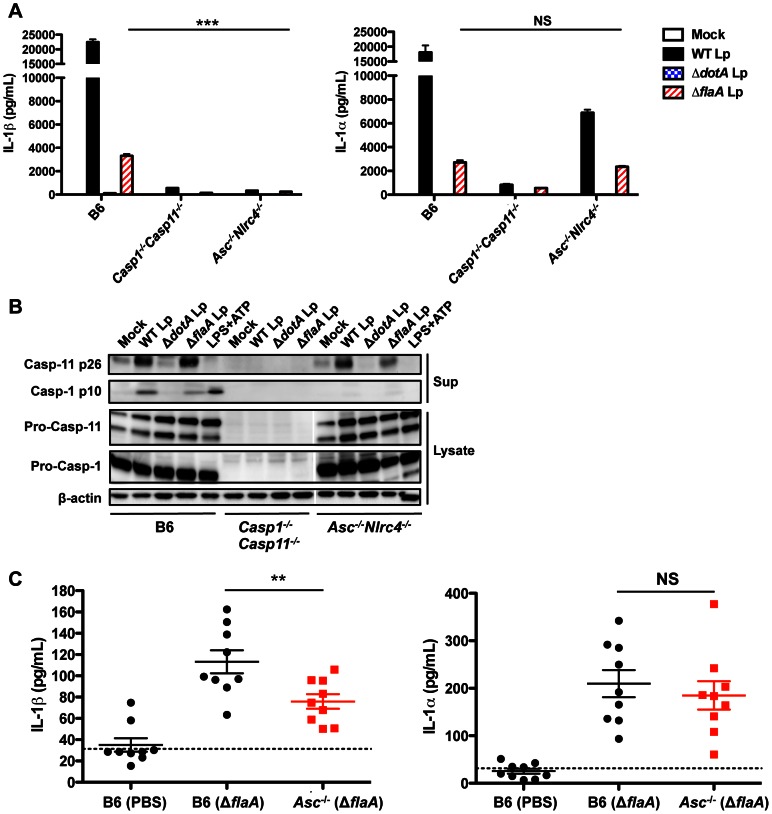
Caspase-11 activation is independent of ASC and NLRC4. (**A**) Unprimed B6, *Casp1^−/−^Casp11^−/−^*, or *Asc^−/−^Nlrc4^−/−^* BMDMs were infected with WT *L. pneumophila* (WT Lp), Δ*dotA* Lp, Δ*flaA* Lp, or PBS (mock infection) for 20 hours, and levels of IL-1α and IL-1β in the supernatants were measured by ELISA. Graphs show the mean ± SEM of triplicate wells. (**B**) Unprimed B6, *Casp1^−/−^Casp11^−/−^*, or *Asc^−/−^Nlrc4^−/−^* BMDMs were infected with WT Lp, Δ*dotA* Lp, Δ*flaA* Lp, or PBS (mock infection) for 20 hours or treated with LPS+ATP for 1 hour. Levels of processed caspase-1 (casp-1 p10) and caspase-11 (casp-11 p26) in the supernatants, and pro-caspase-1, pro-caspase-11, and β-actin (loading control) in the cell lysates were determined by immunoblot analysis. (**C**) 8–12 week old B6 and *Asc^−/−^* mice were infected intranasally with either 1×10^6^ Δ*flaA* Lp or PBS. Bronchoalveolar lavage fluid (BALF) was collected 24 hours post-infection, and levels of IL-1α and IL-1β were measured by ELISA. Graphs show the mean ± SEM of 9 mice per group. Dashed line represents the limit of detection. Data are representative of three independent experiments (A,B) or are displayed as the pooled results of two independent experiments (C). *** is p<0.001 by two-way ANOVA with Bonferroni post-test. ** is p<0.01 by unpaired t-test. NS is not significant.

The ASC and NAIP5/NLRC4 inflammasomes are required for caspase-1 activation and IL-1β secretion in response to *L. pneumophila* [47]. To determine if these inflammasomes are also required for caspase-11 activation and IL-1α release, we infected ASC/NLRC4-deficient (*Asc^−/−^Nlrc4^−/−^*) BMDMs with *L. pneumophila*. *Asc^−/−^Nlrc4^−/−^* BMDMs do not secrete IL-1β in response to either WT Lp, Δ*flaA* Lp, or LPS+ATP. However, *Asc^−/−^Nlrc4^−/−^* BMDMs still release IL-1α in response to Δ*flaA* Lp in primed and unprimed macrophages (Figures 3A, S4). Thus, unlike IL-1β, IL-1α is released independently of flagellin, ASC, and NLRC4. Accordingly, despite an absence of processed caspase-1 p10, robust levels of processed caspase-11 p26 are detected in the supernatants of *Asc^−/−^Nlrc4^−/−^* cells infected with either WT or Δ*flaA* Lp but not in response to LPS+ATP (Figure 3B).

We next sought to determine whether IL-1α is also released independently of ASC and NLRC4 during *in vivo* infection. Because flagellin-deficient *L. pneumophila* do not activate the NLRC4 inflammasome [16], [17], [47], infecting *Asc^−/−^* mice with Δ*flaA* Lp eliminates both the ASC and NLRC4 inflammasome pathways. Importantly, the level of IL-1β in the bronchoalveolar lavage fluid (BALF) 24 hours post-infection is significantly attenuated in *Asc^−/−^* mice infected with Δ*flaA* Lp (Figure 3C). In contrast, the level of IL-1α in the BALF is unaffected even in the absence of both the ASC and NLRC4 inflammasomes. Both IL-1α and IL-1β release are significantly diminished in caspase-1/caspase-11-deficient mice (Figure S5). Collectively, our data indicate that *L. pneumophila* triggers caspase-11 activation and IL-1α release independently of the ASC and NLRC4 inflammasomes during both *in vitro* and *in vivo* infection.

### Caspase-11 mediates both NLRP3-dependent and NLRP3-independent inflammasome responses

**Figure 4:**
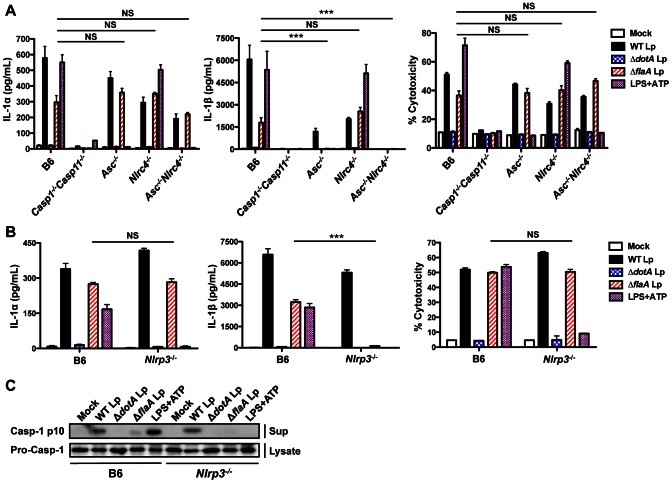
Caspase-11 mediates both NLRP3-dependent and NLRP3-independent immune responses. (**A**) B6, *Casp1^−/−^Casp11^−/−^*, *Asc* ^−/−^, *Nlrc4* ^−/−^, or *Asc^−/−^Nlrc4^−/−^* BMDMs were primed with 0.5 µg/mL LPS for 2.5 hours and infected with WT *L. pneumophila* (WT Lp), Δ*dotA* Lp, Δ*flaA* Lp, or PBS (mock infection) or treated with 2.5 mm ATP for 4 hours. Levels of IL-1α and IL-1β in the supernatants were measured by ELISA and cell death (% cytotoxicity) was measured by LDH release into the supernatants relative to Triton X-100-lysed cells. Graphs show the mean ± SEM of triplicate wells. (**B** and **C**) B6 or *Nlrp3^−/−^* BMDMs were primed with 0.5 µg/mL LPS for 2.5 hours and infected with WT Lp, Δ*dotA* Lp, Δ*flaA* Lp, or PBS (mock infected) or treated with 2.5 mm ATP for 1 hour (C) or 4 hours (B). (B) Levels of IL-1α and IL-1β in the supernatants were measured by ELISA and cell death (% cytotoxicity) was measured by LDH release into the supernatants relative to Triton X-100-lysed cells. Graphs show the mean ± SEM of triplicate wells. (C) Levels of processed caspase-1 (casp-1 p10) in the supernatants and pro-caspase-1 in the cell lysates were determined by immunoblot analysis. Data are representative of two (A,C) or three (B) independent experiments. *** is p<0.001 by one-way ANOVA with Tukey post-test. NS is not significant.

*L. pneumophila* induces caspase-1 activation and IL-1β and IL-18 secretion through two genetically distinct pathways, one involving ASC and one involving NLRC4 (Figures 4A, S6A–B) [47]. The upstream host and bacterial components of the ASC-dependent response to *L. pneumophila* are still unknown, but are independent of the flagellin/NAIP5/NLRC4 pathway (Figures 4A, S6B) [47]. Because caspase-11 contributes to maximal IL-1β secretion in response to Δ*flaA* Lp, we further investigated the ASC-dependent mechanism of inflammasome activation. NLRP3, an NLR involved in inflammasome-dependent responses to a wide variety of pathogens, requires ASC to mediate caspase-1 processing during both canonical and non-canonical inflammasome activation [9], [12], [18], [26], [56]. We therefore investigated the role of NLRP3 in the response to Δ*flaA* Lp. Notably, IL-1β and IL-18 secretion are abrogated during infection of NLRP3-deficient (*Nlrp3* ^−/−^) BMDMs with Δ*flaA* Lp in both primed and unprimed macrophages (Figures 4B, S7A–C). Consistently, we do not detect processed caspase-1 p10 in the supernatants of *Nlrp3* ^−/−^ macrophages infected with Δ*flaA* Lp (Figure 4C). Thus, NLRP3 functions together with ASC, caspase-1, and caspase-11 to control IL-1β secretion in response to flagellin-deficient *L. pneumophila*. However, IL-1α release and cell death following infection with flagellin-deficient *L. pneumophila* are independent of NLRP3 (Figures 4B, S7A), indicating that caspase-11 also mediates an NLRP3-independent response towards flagellin-deficient *L. pneumophila*. Accordingly, NLRP3-dependent IL-1β secretion in response to flagellin-deficient *L. pneumophila* was inhibited by extracellular potassium, whereas NLRP3-independent caspase-11-dependent IL-1α secretion and cell death were not affected (Figure S7D–E).

### Non-canonical inflammasome responses to *L. pneumophila* occur independently of TRIF and IFNAR

**Figure 5:**
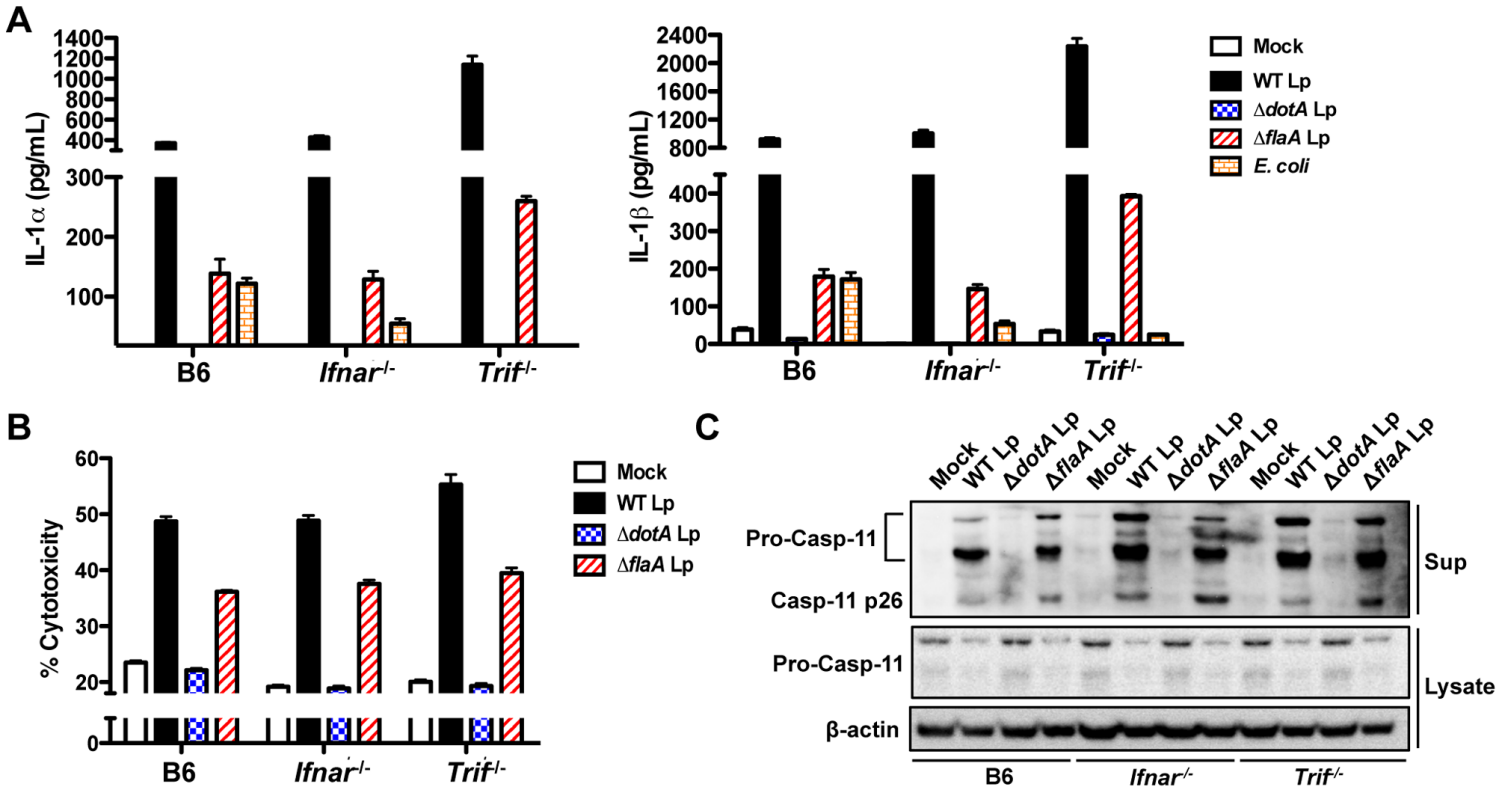
Non-canonical inflammasome responses to *L.* *pneumophila* occur independently of TRIF and IFNAR. (**A**) Unprimed B6, *Ifnar^−/−^*, or *Trif^−/−^* BMDMs were infected with WT *L. pneumophila* (WT Lp), Δ*dotA* Lp, Δ*flaA* Lp, *E. coli*, or PBS (mock infection) for 16 hours. Levels of IL-1α and IL-1β in the supernatants were measured by ELISA. (**B**) Unprimed B6, *Ifnar^−/−^*, or *Trif^−/−^* BMDMs were infected with WT Lp, Δ*dotA* Lp, Δ*flaA* Lp, or PBS (mock infection) for 16 hours. Cell death (% cytotoxicity) was measured by LDH release into the supernatants relative to Triton X-100-lysed cells. Graphs show the mean ± SEM of triplicate wells. (**C**) B6, *Ifnar^−/−^*, or *Trif^−/−^* BMDMs were primed with 0.4 µg/mL Pam3CSK4 for 4 hours and infected with WT Lp, Δ*dotA* Lp, Δ*flaA* Lp, or PBS for 16 hours. Levels of full-length caspase-11 (pro-casp-11) and processed caspase-11 (casp11 p26) in the supernatants and pro-casp-11 and β-actin (loading control) in the cell lysates were determined by immunoblot analysis. Data are representative of two independent experiments.

Recent data demonstrate that caspase-11 activation in response to a wide variety of Gram-negative bacteria requires TLR4 signaling through its adaptor TRIF and subsequent type I IFN production [27]–[29]. To determine if *L. pneumophila* engages a similar TRIF and type I IFN receptor (IFNAR)-dependent pathway for caspase-11 activation, we infected TRIF-deficient (*Trif* ^−/−^) and IFNAR-deficient (*Ifnar* ^−/−^) BMDMs. Unlike the response to *E. coli*, *L. pneumophila* infection of unprimed macrophages triggered robust cell death and secretion of IL-1α and IL-1β that was independent of IFNAR and TRIF (Figure 5A–B). Consistently, priming with the TLR1/2 agonist Pam3CSK4, which results in TRIF- and IFNAR-dependent cytokine secretion and cell death in response to *E. coli* [27], still induced cell death and cytokine secretion in TRIF- and IFNAR-deficient cells in response to *L. pneumophila* (Figure S8A–B). These data suggest that during *L. pneumophila* infection, caspase-11 is upregulated and activated independently of TRIF and IFNAR signaling. Indeed, caspase-11 is still robustly processed and secreted independently of IFNAR and TRIF (Figures 5C, S9). Notably, substantially upregulated levels of pro-caspase-11 are not observed in the lysates of cells infected with WT or Δ*flaA* Lp because both the pro and cleaved forms of caspase-11 are rapidly secreted into the cell supernatant upon infection (Figures 5C, S9). Accordingly, lysates from IFNAR- and TRIF-deficient macrophages infected with *L. pneumophila* express comparable levels of pro-caspase-11 to wild-type macrophages, whereas TRIF and IFNAR do contribute to upregulation of pro-caspase-11 in response to *E. coli* (Figure S10A–C). When the macrophages are primed with LPS prior to infection, there is a moderate contribution of TRIF and IFNAR signaling to inflammasome activation, consistent with the observation that LPS stimulates the TLR4-TRIF-IFNAR axis involved in caspase-11 upregulation (Figure S8C–D). Because the caspase-11-dependent response to *L. pneumophila* is TRIF-independent, we investigated whether the TLR signaling adaptor MyD88 contributes to caspase-11 upregulation. When immortalized macrophages deficient for both MyD88 and Trif (i*Myd88^−/−^Trif^−/−^*) were infected, caspase-11 upregulation was abrogated in response to both WT and Δ*flaA* Lp (Figure S11A–B), and we were unable to detect caspase-11 activation (data not shown). Thus, although TRIF is not required for caspase-11 activation, a TLR-dependent signal is likely required as the loss of both MyD88 and TRIF eliminates caspase-11 upregulation and activation.

### Caspase-11 mediates inflammasome activation in response to *Yersinia pseudotuberculosis* type III secretion system activity

**Figure 6:**
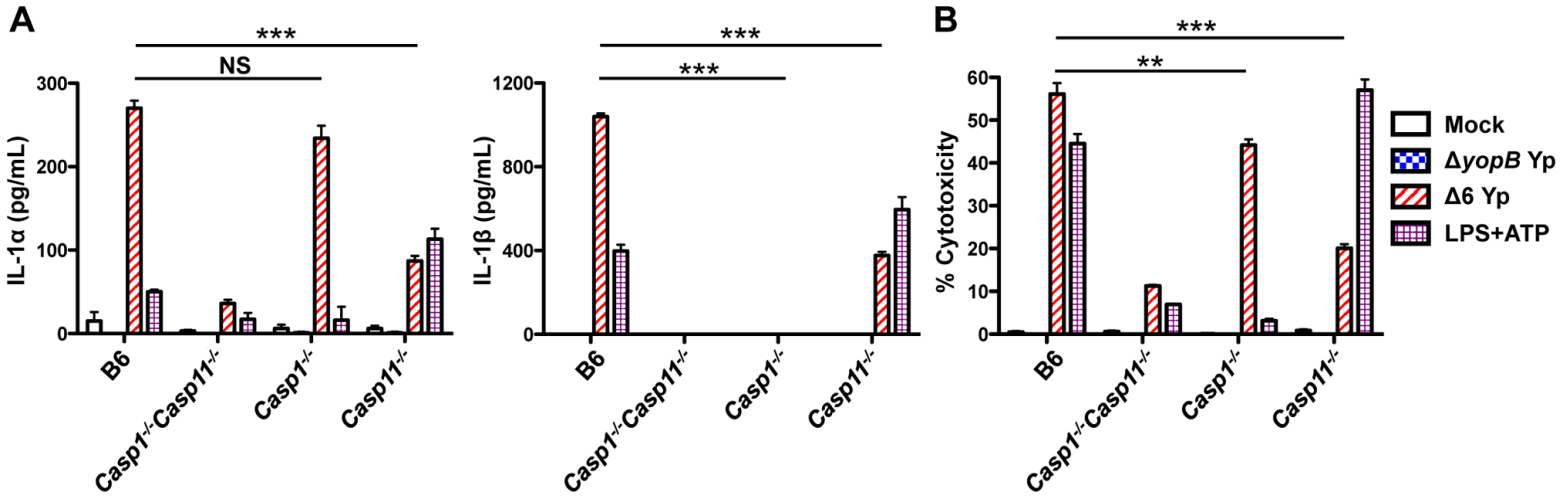
Caspase-11 mediates inflammasome activation in response to a functional *Yersinia* type III secretion system. BMDMs from B6, *Casp1^−/−^Casp11^−/−^*, *Casp1^−/−^*, or *Casp11^−/−^* mice were primed with 0.05 µg/mL LPS for 4 hours and infected with type III secretion system-deficient *Y. pseudotuberculosis* (Δ*yopB* Yp), effectorless *Y. pseudotuberculosis* ΔHOJMEK (Δ6 Yp), or PBS (mock infection) or treated with 2.5 mm ATP for 4 hours. (**A**) Levels of IL-1α and IL-1β in the supernatants were measured by ELISA. (**B**) Cell death (% cytotoxicity) was measured by lactate dehydrogenase (LDH) release relative to Triton X-100-lysed cells. Graphs show the mean ± SEM of triplicate wells. Data are representative of two independent experiments. *** is p<0.001 and ** is p<0.01 by two-way ANOVA with Bonferroni post-test. NS is not significant.

Because caspase-11 activation in response to *L. pneumophila* expressing a functional T4SS is so rapid and robust, we sought to test whether this robust caspase-11-dependent inflammasome activation might be a general response to the activity of specialized secretion systems that allow for bacterial access to the host cytosol. The *Yersinia pseudotuberculosis* type III secretion system (T3SS) induces inflammasome activation independently of bacterial flagellin and the known secreted effector proteins, and this inflammasome activation is important for bacterial clearance [57]. Since wild-type *Yersinia* induces cell death that is independent of both caspase-1 and -11 and requires the secreted effector YopJ [57], [58], we instead infected *Casp1* ^−/−^ *Casp11* ^−/−^, *Casp1* ^−/−^, and *Casp11* ^−/−^ BMDMs with a strain of *Y. pseudotuberculosis* that expresses a T3SS but lacks the six known secreted effectors (Δ6 Yp). Similarly to *L. pneumophila* infection, both IL-1α and IL-1β release in response to Δ6 Yp are caspase-11-dependent (Figure 6A). Again, caspase-1 is absolutely required for IL-1β secretion, whereas IL-1α is released independently of caspase-1. Secretion of IL-12, an inflammasome-independent cytokine, is unaffected (Figure S12). Cell death in response to Δ6 Yp is both caspase-1 and caspase-11-dependent, with a more dramatic reduction in death in *Casp11* ^−/−^ BMDMs (Figure 6B). Furthermore, *Y. pseudotuberculosis*-induced release of both IL-1α and IL-1β requires the presence of a functional T3SS, as *Y. pseudotuberculosis* unable to form a functional T3SS pore in the host cell plasma membrane (Δ*yopB* Yp) do not induce secretion of either cytokine. These data indicate a general role for caspase-11 in the induction of rapid cell death and robust release of IL-1α and IL-1β in response to bacterial secretion systems that are capable of accessing the host cell cytosol, but may be independent of the activities of specific virulence factors per se.

### IL-1α and IL-1β control bacterial burden and neutrophil recruitment *in vivo*

**Figure 7:**
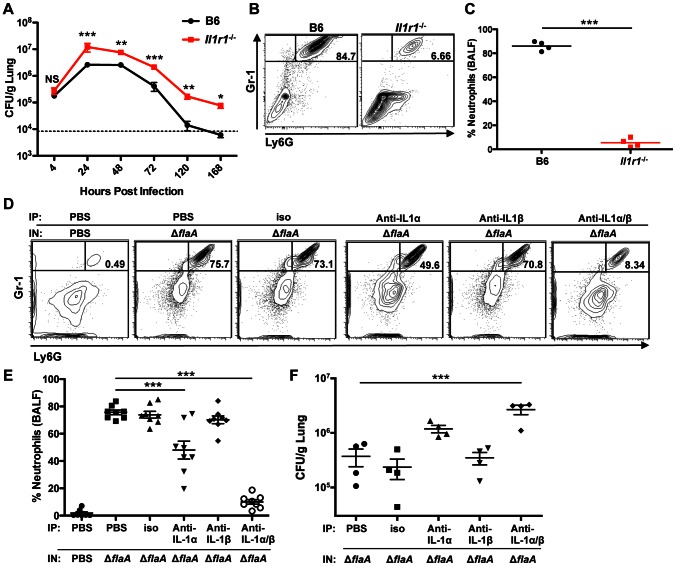
IL-1α and IL-1β control bacterial burden and neutrophil recruitment *in vivo*. (**A**) 8–12 week old B6 or *Il1r1^−/−^* mice were infected with 1×10^6^ Δ*flaA L. pneumophila* intranasally (IN). Lungs were plated to quantify CFU per gram. Graph shows the mean ± SEM of three or four infected mice per group. Dashed line represents the limit of detection. (**B** and **C**) B6 or *Il1r1^−/−^* mice were infected with 1×10^6^ Δ*flaA* Lp IN. 24 hours post-infection, bronchoalveolar lavage fluid (BALF) was collected and the percentage of neutrophils in the BALF was quantified by flow cytometry. Percentages are reported as the frequency of live cells in the BALF. (B) Representative flow cytometry plots showing the percentage of Gr-1^+^Ly6G^+^ neutrophils. (C) Graph showing the percentage of neutrophils. Each point represents an individual mouse and lines indicate the mean of 4 mice per group. (**D, E**, and **F**) B6 mice were injected intraperitoneally (IP) with either PBS, 100 µg isotype control antibody (iso), 100 µg anti-IL-1α antibody, 100 µg anti-IL-1β antibody, or 100 µg each of anti-IL-1α and anti-IL-1β (anti-IL-1α/β) 16 hours before infection. The mice were then intranasally infected with either 1×10^6^ Δ*flaA* Lp or mock infected with PBS. (D and E) 24 hours post-infection, BALF was collected and flow cytometry was performed to quantify the percentage of neutrophils. (D) Representative flow cytometry plots showing the percentage of Gr-1^+^Ly6G^+^ neutrophils. (E) Graph showing the percentage of neutrophils. Each point represents an individual mouse, lines indicate the mean of 8 mice per group, and error bars represent SEM. Shown are the pooled results of two independent experiments. (F) 72 hours post-infection, the lungs were plated to quantify CFU per gram. Each point represents an individual mouse. Line indicates the mean of 4 infected mice per group with error bars representing SEM. *** is p<0.001 by one-way ANOVA with Tukey post-test or unpaired t-test (C). **is p<0.01 and *is p<0.05 by unpaired t-test. NS is not significant.

As caspase-11 contributes to flagellin-independent IL-1α and IL-1β release from infected macrophages *in vitro* and IL-1α and IL-1β secretion is flagellin-independent *in vivo*, we wanted to determine the contribution of IL-1α and IL-1β to host defense against *L. pneumophila in vivo*. IL-1α and IL-1β both bind the IL-1 receptor (IL-1R), which signals through the MyD88 adaptor protein [59]–[61]. As MyD88 is critical for control of *L. pneumophila* replication during *in vivo* infection but deletion of an individual MyD88-dependent TLR or a combination of TLRs does not recapitulate MyD88 deficiency, it is likely that other MyD88-dependent receptors, including the IL-1R, may play a role [62]–[66]. IL-1R signaling contributes to chemokine production by non-hematopoietic cells during infection with wild-type, flagellin-expressing *L. pneumophila* [67]. However, the role of IL-1R signaling during infection with flagellin-deficient *L. pneumophila*, which do not activate the NAIP5/NLRC4 inflammasome, has not been investigated. We therefore infected B6 and IL-1R-deficient (*Il1r1^−/−^*) mice intranasally with Δ*flaA* Lp and measured bacterial burden in the lung over the course of seven days. Though both B6 and *Il1r1^−/−^* mice received similar initial bacterial burdens, *Il1r1^−/−^* mice show a defect in bacterial clearance as early as 24 hours post-infection (Figure 7A). Bacterial burden remains elevated in the absence of IL-1R signaling, with the *Il1r1^−/−^* mice still exhibiting a log-increase in bacterial load at 120 hours post-infection. Since IL-1R signaling is important for neutrophil recruitment [68], we examined whether *Il1r1^−/−^* mice have a defect in neutrophil recruitment to the pulmonary airway during *L. pneumophila* infection. Indeed, *Il1r1^−/−^* mice exhibit a significant decrease in neutrophil recruitment to the airway 24 hours post-infection, possibly contributing to their inability to efficiently clear the pathogen (Figure 7B–C).

The IL-1R signals in response to both IL-1α and IL-1β; however, these cytokines can play non-redundant roles in anti-bacterial defense [69]. To determine the relative contributions of IL-1α and IL-1β to neutrophil recruitment and bacterial clearance during *L. pneumophila* infection, we utilized neutralizing antibodies to selectively block either IL-1α or IL-1β prior to infection. Specific cytokine neutralization in the BALF could be observed 24 hours post-infection (Figure S13). Critically, IL-1α neutralization alone significantly diminishes the percentage of neutrophils recruited to the BALF at 24 hours post-infection and results in a half-log increase in bacterial CFUs, in marked contrast to isotype control antibody or neutralization of IL-1β, which on its own did not have a significant effect (Figure 7D–F). However, neutralization of both IL-1α and IL-1β fully recapitulates the magnitude of neutrophil reduction and defect in bacterial clearance observed in the *Il1r1^−/−^* mice. Collectively, these data indicate that although there are some overlapping roles for these cytokines during *L. pneumophila* infection, IL-1α plays a distinct role from IL-1β in driving neutrophil recruitment to the airway and mediating bacterial clearance.

## Discussion

**Figure 8:**
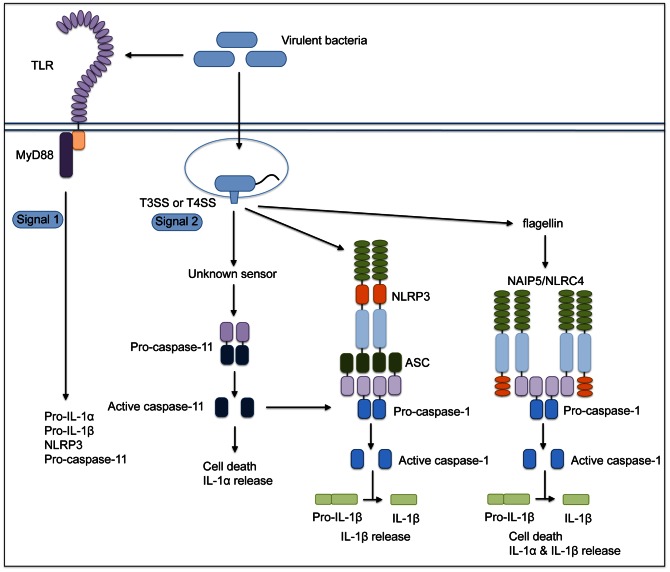
Caspase-11 controls multiple pathways of inflammasome activation in response to bacterial secretion systems that access the host cytosol. Three distinct inflammasome pathways are induced upon interaction of virulent bacteria with host cells. Translocation of flagellin into the host cytosol by specialized secretion systems triggers a NAIP5/NLRC4/caspase-1 inflammasome that leads to cell death, IL-1α, and IL-1β release. Virulent bacteria induce two separate pathways of caspase-11-dependent inflammasome activation through a two-signal model. First, TLR stimulation by PAMPs (signal one) leads to upregulation of pro-IL-1α, pro-IL-1β, NLRP3, and pro-caspase-11. Next, cytosolic detection of virulence activity, namely type III or type IV secretion (signal two), leads to caspase-11 processing and activation. Active caspase-11 contributes to NLRP3-mediated inflammasome activation and caspase-1-dependent IL-1β secretion. Caspase-11 also mediates caspase-1-independent cell death and IL-1α release through a pathway that is independent of the NLRP3/ASC and NAIP5/NLRC4 inflammasomes and involves an unknown host sensor.

Inflammasomes respond robustly to conserved features of pathogenic microbes, such as pore-forming toxins or specialized secretion systems that access the host cytosol. Inflammasomes therefore play a central role in enabling the immune system to discriminate between virulent and avirulent bacteria [70]. Recent reports show a role for caspase-11 in regulating the activation of a non-canonical inflammasome that promotes cell death as well as IL-1α and IL-1β secretion. This non-canonical inflammasome responds to both pathogenic and non-pathogenic Gram-negative bacteria independently of specialized secretion systems that translocate bacterial molecules into the host cytosol [26]–[29]. This pathway involves the TRIF- and IFNAR-dependent upregulation and activation of caspase-11 and occurs with relatively delayed kinetics in comparison to the response to pathogenic bacteria. Intriguingly, we find that the activity of the *L. pneumophila* Dot/Icm T4SS leads to rapid and robust caspase-11 activation independently of the TRIF-IFNAR axis, and this activation triggers rapid cell death and release of both IL-1α and IL-1β (Figure 8). We extend these results to show that the evolutionarily distinct T3SS of another pathogen, *Y. pseudotuberculosis*, also rapidly triggers caspase-11-dependent responses. Collectively, our findings demonstrate that caspase-11 is critical for inflammasome activation in response to the secretion systems of virulent bacteria that enable bacterial molecules to access the host cell cytosol and demonstrate that IL-1α and IL-1β together play a crucial protective role during acute infection *in vivo*.

We demonstrate that in response to the activity of bacterial secretion systems that enable cytosolic access, caspase-11 contributes to NLRP3-mediated inflammasome activation and caspase-1-dependent IL-1β secretion and to a second ASC and NLRC4-independent pathway that does not require caspase-1 and leads to cell death as well as robust IL-1α release. These *L. pneumophila*-induced pathways are similar to recent findings with a number of Gram-negative bacterial pathogens, including *C. rodentium*, *E. coli*, and *S. typhimurium* [26]–[29]. However, we observe rapid and robust T4SS-dependent activation of these two caspase-11-mediated pathways by *L. pneumophila*, whereas the response to Gram-negative bacteria lacking specialized secretion systems occurs less robustly and with much slower kinetics. Intriguingly, we observe a similarly rapid caspase-11-dependent induction of cell death and IL-1 release in response to the structurally and evolutionarily unrelated T3SS of *Y. pseudotuberculosis*. Importantly, this pathway is independent of host sensing of flagellin, as it is triggered by flagellin-deficient *L. pneumophila*, and *Y. pseudotuberculosis* downregulates flagellin expression when the T3SS is expressed [71]. Thus, our data suggest that the caspase-11 inflammasome is poised to respond robustly and rapidly to the activity of bacterial secretion systems that are capable of delivering microbial products to the host cell cytosol and may enable the host to respond to pathogens that evade flagellin-dependent responses. This could have significance for understanding the role of caspase-11 activation at mucosal sites colonized by large numbers of commensal bacteria. At mucosal barriers, it would be expected that the non-canonical inflammasome pathway would not be robustly activated by commensal bacteria but could respond rapidly to the presence of bacterial secretion systems that enable pathogen access to the host cytosol.

Our findings are consistent with recent observations that the *L. pneumophila* Dot/Icm T4SS triggers the caspase-11-dependent non-canonical inflammasome [72], as well as the finding that bacteria that enter the cytosol either due to failure to maintain integrity of their replicative vacuoles or natural entry into the cytoplasm also trigger rapid caspase-11 activation [73]. Thus the pathway that leads to caspase-11 activation appears to be particularly sensitive to pathogens that ‘violate the sanctity of the cytosol’ [74], either through the activity of specialized secretion systems that translocate bacterial molecules into the cytosol or through their direct entry into the host cell cytosol. Whether other pathogens that replicate within the cytosol, such as *Listeria* or *Shigella*, or cytosolic viruses possess mechanisms to evade this pathway remains to be determined.

*L. pneumophila* T4SS-mediated activation of caspase-11 differs from the other pathways of non-canonical inflammasome activation in several ways. First, *L. pneumophila*-mediated activation of caspase-11 does not require TRIF or IFNAR signaling. We observe a moderate dependence on TRIF and IFNAR signaling when macrophages are primed with LPS prior to infection, consistent with LPS-dependent upregulation of caspase-11 expression through the TLR4-TRIF-IFNAR axis [27]–[29]. However, in the absence of LPS priming, TRIF and IFNAR signaling are dispensable for *L. pneumophila*-dependent caspase-11 activation. In this context, it is likely that MyD88 compensates for the absence of TRIF, as cells deficient for both MyD88 and TRIF failed to activate caspase-11 in response to *L. pneumophila*. Thus, although the TLR4-TRIF-IFNAR axis is required for caspase-11 activation in response to Gram-negative bacteria, a MyD88-dependent signal is sufficient for caspase-11 activation in response to pathogens that utilize virulence-associated secretion systems to translocate bacterial molecules into the host cytosol. It is possible that different signals are capable of activating caspase-11 through distinct pathways, but these pathways occur with distinct kinetics because they may indicate distinct levels of pathogenicity. Thus, while caspase-11 is robustly upregulated by LPS priming, this upregulation alone is insufficient for rapid activation in response to bacteria that lack specialized secretion systems, as Δ*dotA* or Δ*yopB* bacteria do not induce rapid cell death even in primed cells. Collectively, these data indicate a two-signal model for rapid caspase-11 activation during infection with virulent bacteria, where bacterial PAMPs induce caspase-11 upregulation, but rapid caspase-11 activation requires a second, secretion system-dependent signal (Figure 8).

The specific secretion system-dependent signals responsible for caspase-11 activation are currently unknown. While rapid activation of caspase-11 requires the presence of a functional type III or type IV secretion system or cytosolic access of the bacteria, whether the signal is an as-yet-undefined translocated bacterial molecule or a cellular response to the pore forming activity of these systems remains to be determined. The delayed NLRP3- and caspase-11-dependent response to Gram-negative bacteria suggests that in addition to LPS-induced upregulation of inflammasome components, bacterial mRNA provides an additional signal for activating the NLRP3 inflammasome [18], [75], although the role of caspase-11 in this response has not been formally demonstrated. Activity of the type III or IV secretion systems may bypass the need for bacterial mRNA. Alternatively, these secretion systems may translocate bacterial RNA [70], [76], [77], and the rapid caspase-11-dependent response they induce could be due to more rapid delivery of bacterial mRNA into the host cell cytosol.

Furthermore, the host factors required for activation of the NLRP3-independent caspase-11-dependent inflammasome also remain to be identified. As this pathway is independent of flagellin sensing, NLRP3, ASC, and NLRC4, an unknown upstream sensor and/or adaptor may be involved in caspase-11 activation in response to a translocated bacterial substrate or an endogenous signal induced by infection. This sensor may also be upregulated by type I IFN signaling itself [27]–[29].

Our data show that IL-1α release during *L. pneumophila* infection is controlled by two independent pathways, one involving the flagellin-dependent NAIP5/NLRC4 and caspase-1-dependent inflammasome and a second pathway involving the NLRP3-independent caspase-11-dependent inflammasome (Figure 8). Though we demonstrate that IL-1α release has an important biological consequence *in vivo* for neutrophil recruitment and bacterial clearance, it is unclear if IL-1α release is regulated by unconventional secretion, as is the case for IL-1β [78]. As both pathways that control IL-1α release also lead to cell death, our data are consistent with a model in which IL-1α is an endogenous alarmin that is released during cell death [79].

Interestingly, caspase-11 also contributes to control of flagellin-expressing *L. pneumophila* by serving as a component of an NLRC4-dependent inflammasome that promotes trafficking of the *L. pneumophila*-containing vacuole to lysosomes [80]. Thus, caspase-11 may function in multiple ways to control *L. pneumophila* infection. Importantly, we find that IL-1α, IL-1β, and IL-1R signaling play an important role in the control of *L. pneumophila* infection through efficient neutrophil recruitment to the airway. IL-1α and IL-1β play both distinct and overlapping roles in mediating neutrophil recruitment and controlling bacterial replication, as depletion of IL-1α alone showed a more pronounced defect in neutrophil recruitment and bacterial clearance than depletion of IL-1β alone, but loss of both cytokines resulted in a further reduction of neutrophil recruitment and an increased defect in bacterial clearance. Further analysis is required to define the relative contributions of the various caspase-11-mediated effector functions to the control of *L. pneumophila* replication *in vivo*.

In conclusion, these studies demonstrate that T3SS and T4SS activities trigger rapid and robust activation of caspase-11. This activation contributes to maximal NLRP3-dependent IL-1β secretion as well as to NLRP3-independent IL-1α release and host cell death. The downstream effector functions of these pathways are important for host defense against *L. pneumophila in vivo*, as IL-1α and IL-1β promote neutrophil recruitment to *L. pneumophila*-infected lungs and control pulmonary bacterial replication. Our results highlight the contribution of caspase-11 to rapid inflammasome activation and discrimination between pathogenic and nonpathogenic bacteria.

## Materials and Methods

### Ethics statement

This study was carried out in strict accordance as defined in the federal regulations set forth in the Animal Welfare Act (AWA), the recommendations in the Guide for the Care and Use of Laboratory Animals of the National Institutes of Health, and the guidelines of the University of Pennsylvania Institutional Animal Use and Care Committee. The protocols were approved by the Institutional Animal Care and Use Committee at the University of Pennsylvania (protocols #803465 and #803459).

### Bacterial strains

*Legionella pneumophila* serogroup 1 strains were used in all experiments. Macrophages were infected with Lp02 (*thyA*), a thymidine auxotroph derived from strain Lp01 [40], or Δ*dotA* [81] and Δ*flaA* [16] isogenic mutant strains. For *in vivo* studies, mice were infected with the Lp02 Δ*flaA* or the JR32 [82] Δ*flaA* isogenic mutant strain where indicated. For *in vitro* and *in vivo* studies, *L. pneumophila* were cultured on charcoal yeast extract agar for 48 hours at 37°C prior to infection. *Escherichia coli* BL21 strain was cultured in LB broth for 16 hours at 37°C prior to infection. The *Yersinia pseudotuberculosis* strains used were IP2666 Δ*yopHOJEMK* (Δ6) [58] and Δ*yopB* [83]. *Yersinia* were grown overnight with aeration in 2×YT broth at 26°C. The bacteria were diluted into fresh 2×YT containing 20 mM sodium oxalate and 20 mM MgCl_2_. Bacteria were grown with aeration for 1 hour at 26°C followed by 2 hours at 37°C prior to infection.

### Mice

C57BL/6 mice were purchased from Jackson Laboratories. *Casp1^−/−^Casp11^−/−^* [84], *Casp1^−/−^* (unpublished data, T.S. and R.A.F.), *Casp11^−/−^* [30], *Asc^−/−^* [23], *Nlrc4^−/−^* [85], *Asc^−/−^Nlrc4^−/−^* [47], *Ifnar^−/−^* [86], *Trif^−/−^* [87], *Il1r1^−/−^* [88], and *Nalp3^−/−^* [89] mice are all on the C57BL/6 background. *Asc^−/−^*, *Nlrc4^−/−^*, and *Nlrp3^−/−^* mice were originally generated by Millenium Pharmaceuticals and were a kind gift of Richard Flavell. Animals were maintained in accordance with the guidelines of the University of Pennsylvania Institutional Animal Use and Care Committee.

### *In vivo* infection studies

8–12 week-old mice were anesthetized by intraperitoneal injection of a ketamine/xylazine/PBS solution at a dose of 100 mg/kg ketamine and 10 mg/kg xylazine. Mice were infected intranasally with 40 µl of a bacterial suspension containing 1×10^6^ CFU *L. pneumophila* or PBS vehicle control. For antibody neutralization experiments, mice were injected intraperitoneally with 100 µg anti-IL-1α antibody (clone ALF-161), 100 µg anti-IL-1β antibody (clone B122), 100 µg of each anti-IL-1α and anti-IL-1β antibody, or 100 µg Armenian hamster IgG_1_ isotype control antibody (eBioscience) 16 hours prior to intranasal infection. At the indicated timepoints after infection, mice were sacrificed, and the bronchoalveolar lavage fluid (BALF) and lungs were harvested. To determine bacterial load, the lungs were mechanically homogenized in sterile distilled H_2_O and a portion of the lysate was spread onto CYE plates. Animal experiments were performed in accordance with approved University of Pennsylvania Institutional Animal Care and Use Committee protocols and procedures.

### Macrophage experiments

Bone marrow was collected from the femurs and tibiae of mice. Bone marrow cells were differentiated into macrophages by culturing the cells in RPMI containing 30% L929 cell supernatant and 20% FBS at 37°C in a humidified incubator. The macrophages were replated one day prior to infection in RPMI containing 15% L929 cell supernatant and 10% FBS. For experiments involving LPS-primed macrophages, macrophages in 48-well plates (2.0×10^5^ cells/well) were pretreated with 0.5 µg/mL LPS for 2.5 hours and either mock-infected with PBS, infected with *L. pneumophila* at an MOI = 10 for 4 hours, or treated with 2.5 mM ATP for 1 or 4 hours. For experiments performed in the absence of LPS priming, macrophages in 48-well plates (2.0×10^5^ cells/well) were either mock-infected with PBS, infected with *L. pneumophila* at an MOI = 10 for 16 or 20 hours, or infected with *E. coli* at an MOI = 25 for 1 hour followed by gentamycin treatment for 15 hours. To assess the involvement of caspase-1 catalytic activity, macrophages were treated with 20 µM or 40 µM of the caspase-1 inhibitor YVAD-cmk (Bachem) or an equivalent volume of dimethyl sulfoxide (vehicle control) 0.5 hours prior to infection. For *L. pneumophila* and *E. coli* infections, bacteria were centrifuged down onto the macrophages at 1200 RPM for ten minutes prior to incubation. For *Y. pseudotuberculosis* infection, bacteria were washed three times with pre- warmed DMEM, added to the cells at an MOI = 20, and centrifuged down onto the macrophages at 1000 rpm for 5 min. Cells were incubated at 37°C for 1 hour post-infection followed by addition of 100 µg/mL gentamicin. Supernatants were harvested 4 hours post infection for ELISA and LDH analysis.

### Cytotoxicity assays

Cells were infected or treated as described above, and supernatants were harvested at the indicated times post-infection. Lactate dehydrogenase (LDH) release was quantified using the LDH Cytotoxicity Assay Kit (Clontech) according to the manufacturer's instructions.

### Immunoblotting

Supernatants from infected cells were mixed 1∶1 with 2 X SDS-PAGE sample buffer or infected BMDMs were directly lysed in 1 X SDS-PAGE sample buffer. Samples were boiled, separated by SDS-PAGE, and transferred to Immobilon P membranes (Millipore). Primary antibodies against caspase-1 p10 (Santa Cruz Biotechnology), caspase-11 (Sigma, clone 17D9), IL-1β (R&D systems), and β-actin (Sigma) were used. Detection was performed with HRP-conjugated anti-rabbit IgG (Cell Signaling Technology) or anti-rat IgG (Santa Cruz Biotechnology or Jackson Immuno).

### ELISA

Harvested supernatants from infected macrophages or the BALF from infected mice were assayed using capture and detection antibodies specific for IL-18 (MBL), IL-1α, IL-1β, and IL-12p40 (BD Biosciences).

### Flow cytometry

To determine neutrophil recruitment to the airway, BALF cells were stained with Live/Dead Fixable Dead Cell Stain (Invitrogen), and antibodies specific for CD45, Gr-1 (eBioscience), and Ly6G (Biolegend). Data were collected with an LSRII flow cytometer (BD Biosciences) and post-collection data was analyzed using FlowJo (Treestar). Cells were gated on singlets and live cells. Neutrophils were identified as being CD45^+^, Gr-1^+^, and Ly6G^+^.

### Statistical analysis

Plotting of data and statistical analysis were performed using Graphpad Prism software, and statistical significance was determined by the unpaired two-tailed Student's t test, one-way ANOVA with Tukey post-test, or two-way ANOVA with Bonferroni post-test. Differences were considered statistically significant if the *P* value was <0.05.

## Supporting Information

Figure S1 **Caspase-1/caspase-11-deficient cells do not have a gross defect in cytokine secretion.** (**A**) Unprimed B6 or *Casp1^−/−^Casp11^−/−^* BMDMs were infected with WT *L. pneumophila* (Lp), Δ*dotA* Lp, Δ*flaA* Lp, or PBS (mock infection) for 20 hours. Levels of full-length IL-1β (pro-IL-1β) and β-actin (loading control) in the cell lysates were determined by immunoblot analysis. (**B**) B6 or *Casp1^−/−^Casp11^−/−^* BMDMs were primed with 0.5 µg/mL LPS for 2.5 hours and infected with WT Lp, Δ*dotA* Lp, Δ*flaA* Lp, or PBS for 4 hours. The level of IL-18 in the supernatants was measured by ELISA. (**C**) Unprimed B6 or *Casp1^−/−^Casp11^−/−^* BMDMs were infected with WT Lp, Δ*dotA* Lp, Δ*flaA* Lp, or PBS for 20 hours. Levels of IL-12 p40 and TNF-α in the supernatants were measured by ELISA. (**D**) B6 or *Casp1^−/−^Casp11^−/−^* BMDMs were primed with 0.5 µg/mL LPS for 2.5 hours and infected with WT Lp, Δ*dotA* Lp, Δ*flaA* Lp, or PBS for 4 hours. Levels of IL-12 p40 and TNF-α in the supernatants were measured by ELISA. Graphs show the mean ± SEM of triplicate wells. Data are representative of two (B) or three (A, C, and D) independent experiments.
(TIF)

Figure S2 **Caspase-11 is rapidly upregulated and secreted in response to** ***L. pneumophila*** **.** B6 or *Casp1^−/−^Casp11^−/−^* BMDMs were primed with 0.5 µg/mL LPS for 2.5 hours and infected with WT *L. pneumophila* (Lp), Δ*dotA* Lp, Δ*flaA* Lp, or PBS (mock infection) for 4 hours. Levels of full-length caspase-11 (pro-casp-11) and active caspase-11 (casp11 p26) in the supernatants, and pro-casp-11 and β-actin (loading control) in the cell lysates were determined by immunoblot analysis.
(TIF)

Figure S3 **Caspase-11 is activated in response to** ***L. pneumophila*** **independently of macrophage priming.** (**A**) Unprimed B6 and *Casp1^−/−^* BMDMs or (**B**) B6, *Casp1^−/−^Casp11^−/−^*, and *Casp11^−/−^* BMDMs were infected with WT *L. pneumophila* (Lp), Δ*dotA* Lp, Δ*flaA* Lp, or PBS (mock infection) for 20 hours. Levels of IL-1α and IL-1β in the supernatants were measured by ELISA. Graphs show the mean ± SEM of triplicate wells. (**C**) B6, *Casp1^−/−^*, or *Casp11^−/−^* BMDMs were primed with 0.5 µg/mL LPS for 2.5 hours and infected with WT Lp, Δ*dotA* Lp, Δ*flaA* Lp, or PBS for 4 hours or treated with LPS+2.5 mm ATP for 1 hour. Levels of mature IL-1β in the supernatant were determined by immunoblot analysis. Data are representative of two independent experiments.
(TIF)

Figure S4 **IL-1α release is ASC/NLRC4-independent.** B6, *Casp1^−/−^Casp11^−/−^*, or *Asc^−/−^Nlrc4^−/−^* BMDMs were primed with 0.5 µg/mL LPS for 2.5 hours and infected with WT *L. pneumophila* (Lp), Δ*dotA* Lp, Δ*flaA* Lp, or PBS (mock infection) or treated with 2.5 mm ATP for 4 hours. Levels of IL-1α and IL-1β in the supernatants were measured by ELISA. Graphs show the mean ± SEM of triplicate wells. Data are representative of three independent experiments.
(TIF)

Figure S5 **Both IL-1α and IL-1β secretion are caspase-1/caspase-11-dependent** ***in vivo*** **.** 8–12 week old B6 or *Casp1^−/−^Casp11^−/−^* mice were infected intranasally with 1×10^6^ Δ*flaA* Lp. BALF was collected 24 hours post-infection, and levels of IL-1α and IL-1β were measured by ELISA. Graphs show the mean ± SEM of three mice per group. Dashed line represents the limit of detection. * is p<0.05 by unpaired t-test.
(TIF)

Figure S6 **Mature IL-1β secretion is not always concordant with cell death.** B6, *Casp1^−/−^Casp11^−/−^*, *Asc* ^−/−^, *Nlrc4* ^−/−^, or *Asc^−/−^Nlrc4^−/−^* BMDMs were primed with 0.5 µg/mL LPS for 2.5 hours and infected with WT *L. pneumophila* (Lp), Δ*dotA* Lp, Δ*flaA* Lp or PBS (mock infection) for 4 hours or treated with 2.5 mm ATP for 1 hour. (**A**) Levels of mature IL-1β in the supernatants, and full-length IL-1β (pro-IL-1β) and β-actin (loading control) in the cell lysates were determined by immunoblot analysis. Data are representative of two independent experiments. (**B**) The level of IL-18 in the supernatants was measured by ELISA. Graphs show the mean ± SEM of triplicate wells.
(TIF)

Figure S7 **Flagellin-independent, NLRP3-dependent IL-1β secretion occurs independently of macrophage priming.** (**A**) Unprimed B6 or *Nlrp3^−/−^* BMDMs were infected with WT *L. pneumophila* (Lp), Δ*dotA* Lp, Δ*flaA* Lp, or PBS (mock infection) for 16 hours. Levels of IL-1α and IL-1β in the supernatants were measured by ELISA. (**B**) B6 or *Nlrp3^−/−^* BMDMs were primed with 0.5 µg/mL LPS for 2.5 hours and infected with WT Lp, Δ*dotA* Lp, Δ*flaA* Lp or PBS (mock infection) or treated with 2.5 mM ATP for 4 hours. The level of IL-18 in the supernatants was measured by ELISA. (**C**) B6 or *Nlrp3^−/−^* BMDMs were infected with WT Lp, Δ*dotA* Lp, Δ*flaA* Lp, or PBS (mock infection) for 16 hours. The level of IL-18 in the supernatants was measured by ELISA. (**D** and **E**) B6 BMDMs were primed with 0.5 µg/mL LPS for 2.5 hours and infected with WT Lp, Δ*dotA* Lp, Δ*flaA* Lp or PBS (mock infection) or treated with 2.5 mM ATP for 4 hours. Where indicated, media alone, 50 mM KCl, or 50 mM NaCl were added prior to infection. (D) Levels of IL-1α and IL-1β in the supernatants were measured by ELISA. (E) Cell death was measured by LDH release. Graphs show the mean ± SEM of triplicate wells. Data are representative of two independent experiments (A–C).
(TIF)

Figure S8 **TRIF/IFNAR-independent IL-1 release occurs with Pam3CSK4-primed macrophages.** (**A**) B6, *Ifnar^−/−^*, or *Trif^−/−^* BMDMs were primed with 0.4 µg/mL Pam3CSK4 for 4 hours and infected with WT *L. pneumophila* (Lp), Δ*dotA* Lp, Δ*flaA* Lp, *E. coli*, or PBS (mock infection) for 16 hours. The levels of IL-1α and IL-1β in the supernatants were measured by ELISA. (**B**) B6, *Ifnar^−/−^*, or *Trif^−/−^* BMDMs were primed with 0.4 µg/mL Pam3CSK4 for 4 hours and infected with WT Lp, Δ*dotA* Lp, Δ*flaA* Lp, or PBS for 16 hours. Cell death (% cytotoxicity) was measured by LDH release. (**C**) B6, *Ifnar^−/−^*, or *Trif^−/−^* BMDMs were primed with 0.5 µg/mL LPS for 2.5 hours and infected with WT Lp, Δ*dotA* Lp, Δ*flaA* Lp, or PBS for 4 hours. Cell death was measured by LDH release. (**D**) B6, *Ifnar^−/−^*, or *Trif^−/−^* BMDMs were primed with 0.5 µg/mL LPS for 2.5 hours and infected with WT Lp, Δ*dotA* Lp, Δ*flaA* Lp, or PBS for 4 hours. Levels of IL-1α and IL-1β in the supernatants were measured by ELISA. Graphs show the mean ± SEM of triplicate wells. Data are representative of two independent experiments.
(TIF)

Figure S9 **Caspase-11 is upregulated and secreted in an IFNAR- and TRIF-independent manner.** Unprimed B6, *Ifnar^−/−^*, or *Trif^−/−^* BMDMs were infected with WT Lp, Δ*dotA* Lp, Δ*flaA* Lp, or PBS for 16 hours. Levels of full-length caspase-11 (pro-casp-11) and active caspase-11 (casp11 p26) in the supernatants, and pro-casp-11 and β-actin (loading control) in the cell lysates were determined by immunoblot analysis.
(TIF)

Figure S10 **Detection of caspase-11 protein upregulation in cell lysates is moderate in response to** ***L. pneumophila*** **.** (**A**) Unprimed B6, *Ifnar^−/−^*, or *Trif^−/−^* BMDMs were infected with WT Lp, Δ*dotA* Lp, Δ*flaA* Lp, *E. coli*, or PBS (mock infection) for 16 hours. (**B**) B6, *Ifnar^−/−^*, or *Trif^−/−^* BMDMs were primed with 0.4 µg/mL Pam3CSK4 for 4 hours and infected with WT Lp, Δ*dotA* Lp, Δ*flaA* Lp, *E. coli*, or PBS for 16 hours. (**C**) B6, *Ifnar^−/−^*, or *Trif^−/−^* BMDMs were primed with 0.5 µg/mL LPS for 2.5 hours and infected with WT Lp, Δ*dotA* Lp, Δ*flaA* Lp, *E. coli*, or PBS for 4 hours. Levels of full-length caspase-11 (pro-casp-11) and β-actin (loading control) in the cell lysates were determined by immunoblot analysis.
(TIF)

Figure S11 **Caspase-11 is not upregulated in the absence of both MyD88 and Trif.** (**A**) Immortalized B6 (iB6) or MyD88/Trif-deficient (i*Myd88^−/−^Trif^−/−^*) BMDMs were primed with 0.4 µg/mL Pam3CSK4 for 4 hours and infected with WT Lp, Δ*dotA* Lp, Δ*flaA* Lp, *E. coli*, or PBS (mock infection) for 16 hours. (**B**) iB6 or i*Myd88^−/−^Trif^−/−^* macrophages were primed with 0.5 µg/mL LPS for 4 hours and infected with WT Lp, Δ*dotA* Lp, Δ*flaA* Lp, or PBS (mock infection) for 4 hours. Levels of full-length caspase-11 (pro-casp-11) and β-actin (loading control) were determined by immunoblot analysis.
(TIF)

Figure S12 **Caspase-11-deficient cells secrete comparable amounts of IL-12 in response to** ***Y. pseudotuberculosis*** **.** B6, *Casp1^−/−^Casp11^−/−^*, *Casp1^−/−^*, or *Casp11^−/−^* mice were primed with 0.05 µg/mL LPS for 2.5 hours and infected with type III secretion system-deficient *Y. pseudotuberculosis* (Δ*yopB* Yp), effectorless *Y. pseudotuberculosis* ΔHOJMEK (Δ6 Yp), or PBS (mock infection) or treated with 2.5 mm ATP for 4 hours. The level of IL-12 p40 in the supernatants was measured by ELISA. Graphs show the mean ± SEM of triplicate wells. Data are representative of two independent experiments.
(TIF)

Figure S13 **Intraperitoneally injected antibodies neutralize cytokine in the BALF.** 8–12 week old B6 mice were injected intraperitoneally (IP) with either PBS, 100 µg anti-IL-1α antibody, 100 µg anti-IL-1β antibody, or 100 µg each of anti-IL-1α and anti-IL-1β (anti-IL-1α/β) 16 hours before infection. The mice were then infected with either 1×10^6^ Δ*flaA* Lp or mock infected with PBS intranasally (IN). 24 hours post-infection, bronchoalveolar lavage fluid (BALF) was collected and the levels of IL-1α and IL-1β were measured by ELISA. Labels indicate what was received intraperitoneally (IP) and what was received intranasally (IN). Graphs show the mean ± SEM of 8 mice per group and represent the pooled results of two independent experiments.
(TIF)
