# Supplementary figures and images for: Caspase-11 Activation in Response to Bacterial Secretion Systems that Access the Host Cytosol
Source: PLoS Pathog. 2013 Jun 6;9(6):e1003400. doi: 10.1371/journal.ppat.1003400 (PMC3675167; doi:10.1371/journal.ppat.1003400)

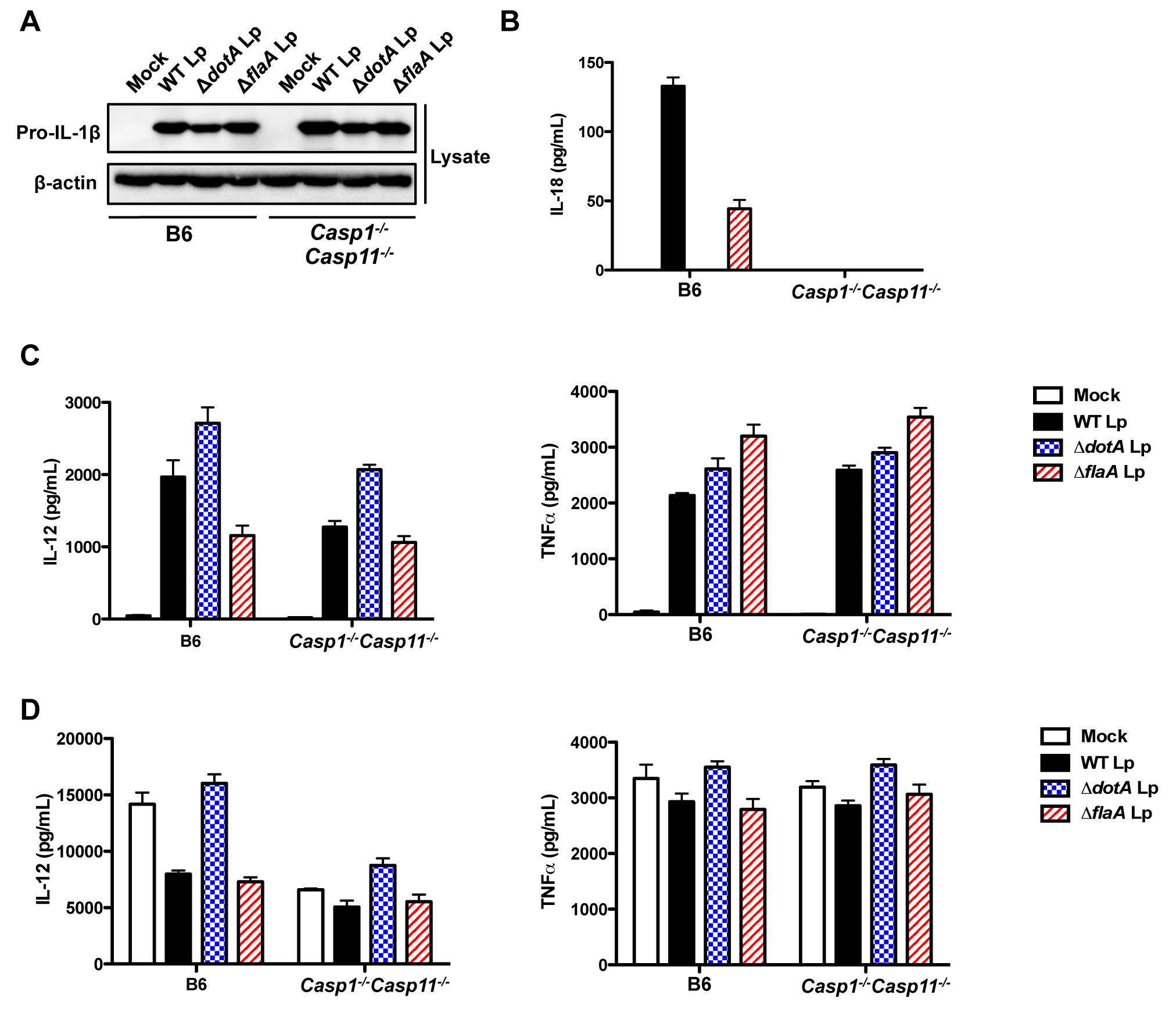

Supplement: Figure S1 — Caspase-1/caspase-11-deficient cells do not have a gross defect in cytokine secretion. (A) Unprimed B6 or Casp1−/−Casp11−/− BMDMs were infected with WT L. pneumophila (Lp), ΔdotA Lp, ΔflaA Lp, or PBS (mock infection) for 20 hours. Levels of full-length IL-1β (pro-IL-1β) and β-actin (loading control) in the cell lysates were determined by immunoblot analysis. (B) B6 or Casp1−/−Casp11−/− BMDMs were primed with 0.5 µg/mL LPS for 2.5 hours and infected with WT Lp, ΔdotA Lp, ΔflaA Lp, or PBS for 4 hours. The level of IL-18 in the supernatants was measured by ELISA. (C) Unprimed B6 or Casp1−/−Casp11−/− BMDMs were infected with WT Lp, ΔdotA Lp, ΔflaA Lp, or PBS for 20 hours. Levels of IL-12 p40 and TNF-α in the supernatants were measured by ELISA. (D) B6 or Casp1−/−Casp11−/− BMDMs were primed with 0.5 µg/mL LPS for 2.5 hours and infected with WT Lp, ΔdotA Lp, ΔflaA Lp, or PBS for 4 hours. Levels of IL-12 p40 and TNF-α in the supernatants were measured by ELISA. Graphs show the mean ± SEM of triplicate wells. Data are representative of two (B) or three (A, C, and D) independent experiments. (TIF) [file ppat.1003400.s001.tif]

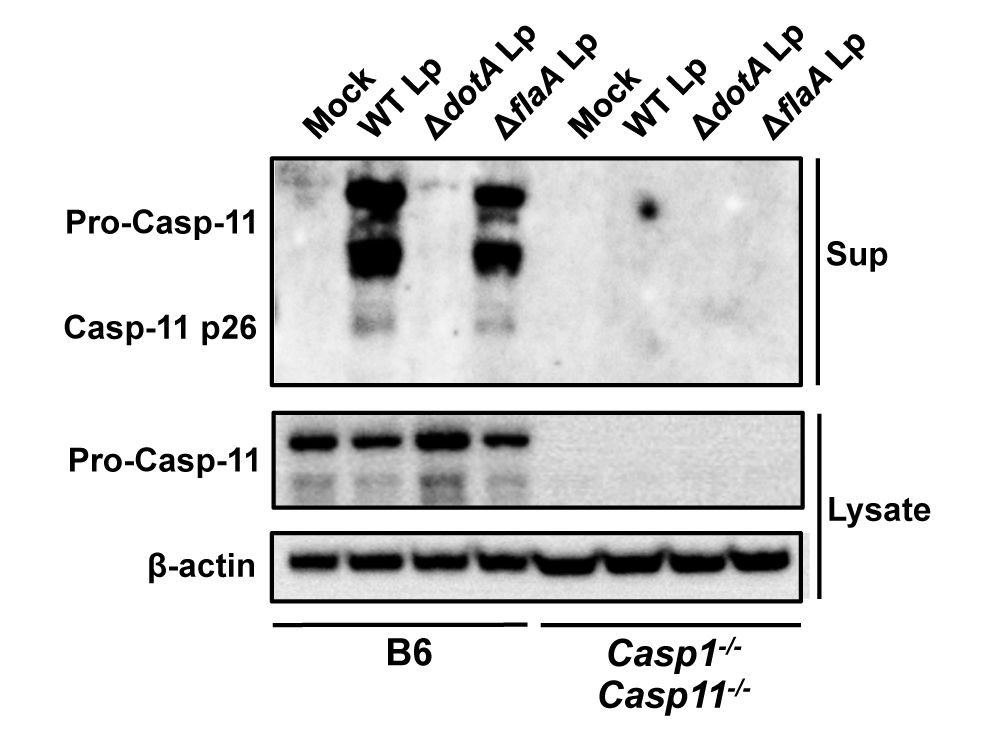

Supplement: Figure S2 — Caspase-11 is rapidly upregulated and secreted in response to L. pneumophila . B6 or Casp1−/−Casp11−/− BMDMs were primed with 0.5 µg/mL LPS for 2.5 hours and infected with WT L. pneumophila (Lp), ΔdotA Lp, ΔflaA Lp, or PBS (mock infection) for 4 hours. Levels of full-length caspase-11 (pro-casp-11) and active caspase-11 (casp11 p26) in the supernatants, and pro-casp-11 and β-actin (loading control) in the cell lysates were determined by immunoblot analysis. (TIF) [file ppat.1003400.s002.tif]

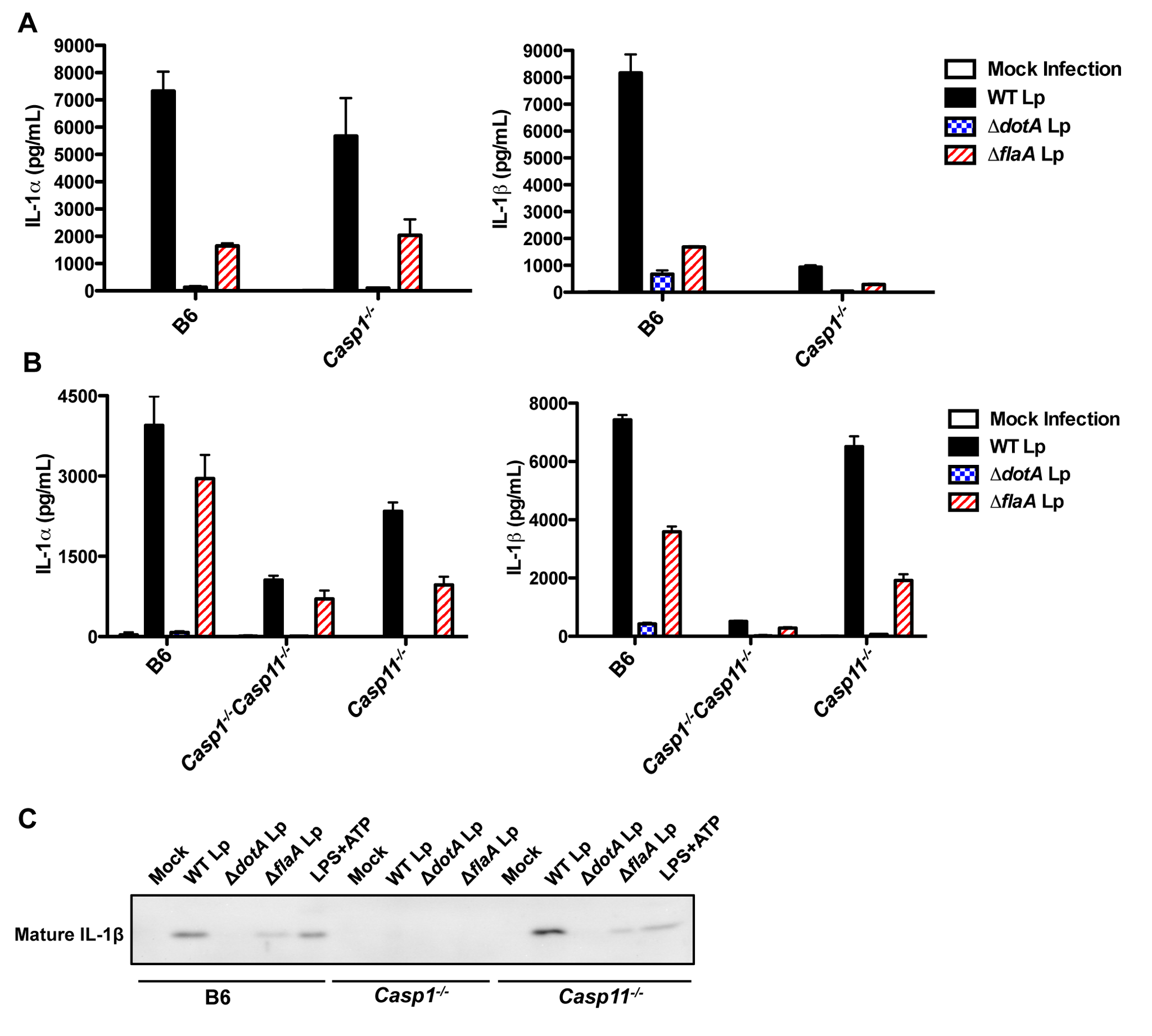

Supplement: Figure S3 — Caspase-11 is activated in response to L. pneumophila independently of macrophage priming. (A) Unprimed B6 and Casp1−/− BMDMs or (B) B6, Casp1−/−Casp11−/−, and Casp11−/− BMDMs were infected with WT L. pneumophila (Lp), ΔdotA Lp, ΔflaA Lp, or PBS (mock infection) for 20 hours. Levels of IL-1α and IL-1β in the supernatants were measured by ELISA. Graphs show the mean ± SEM of triplicate wells. (C) B6, Casp1−/−, or Casp11−/− BMDMs were primed with 0.5 µg/mL LPS for 2.5 hours and infected with WT Lp, ΔdotA Lp, ΔflaA Lp, or PBS for 4 hours or treated with LPS+2.5 mm ATP for 1 hour. Levels of mature IL-1β in the supernatant were determined by immunoblot analysis. Data are representative of two independent experiments. (TIF) [file ppat.1003400.s003.tif]

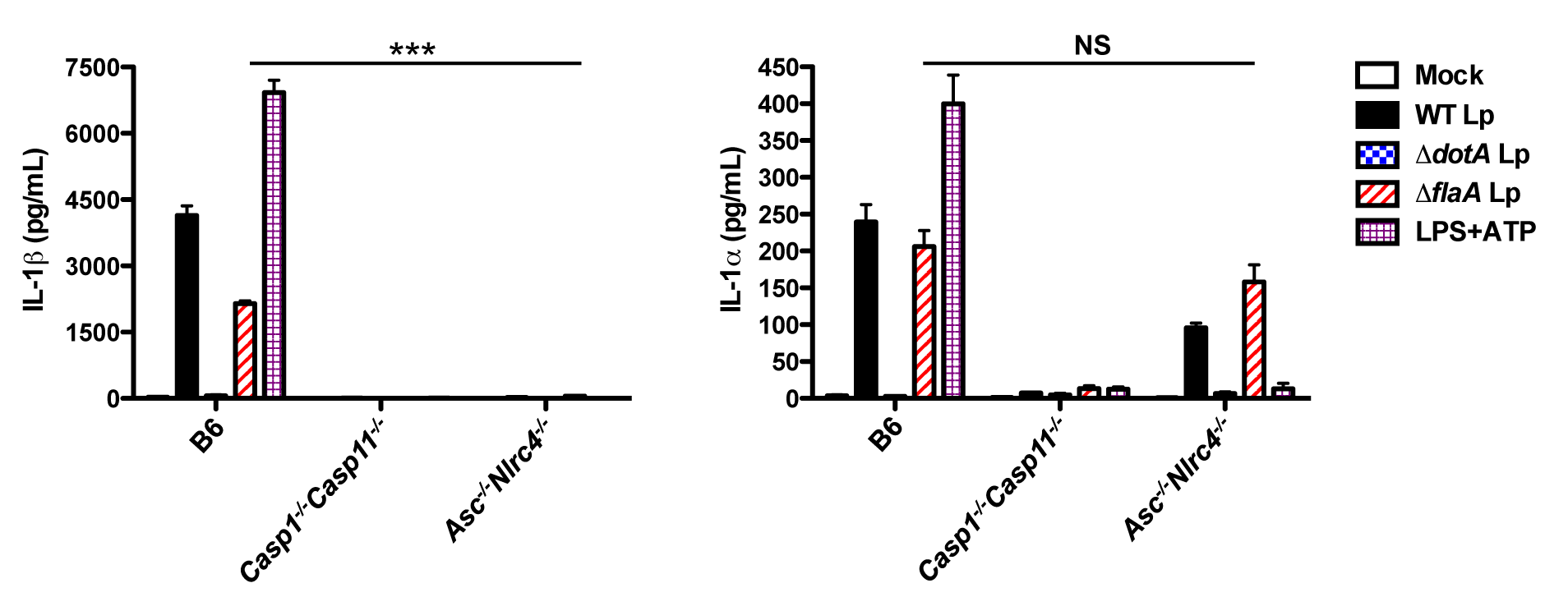

Supplement: Figure S4 — IL-1α release is ASC/NLRC4-independent. B6, Casp1−/−Casp11−/−, or Asc−/−Nlrc4−/− BMDMs were primed with 0.5 µg/mL LPS for 2.5 hours and infected with WT L. pneumophila (Lp), ΔdotA Lp, ΔflaA Lp, or PBS (mock infection) or treated with 2.5 mm ATP for 4 hours. Levels of IL-1α and IL-1β in the supernatants were measured by ELISA. Graphs show the mean ± SEM of triplicate wells. Data are representative of three independent experiments. (TIF) [file ppat.1003400.s004.tif]

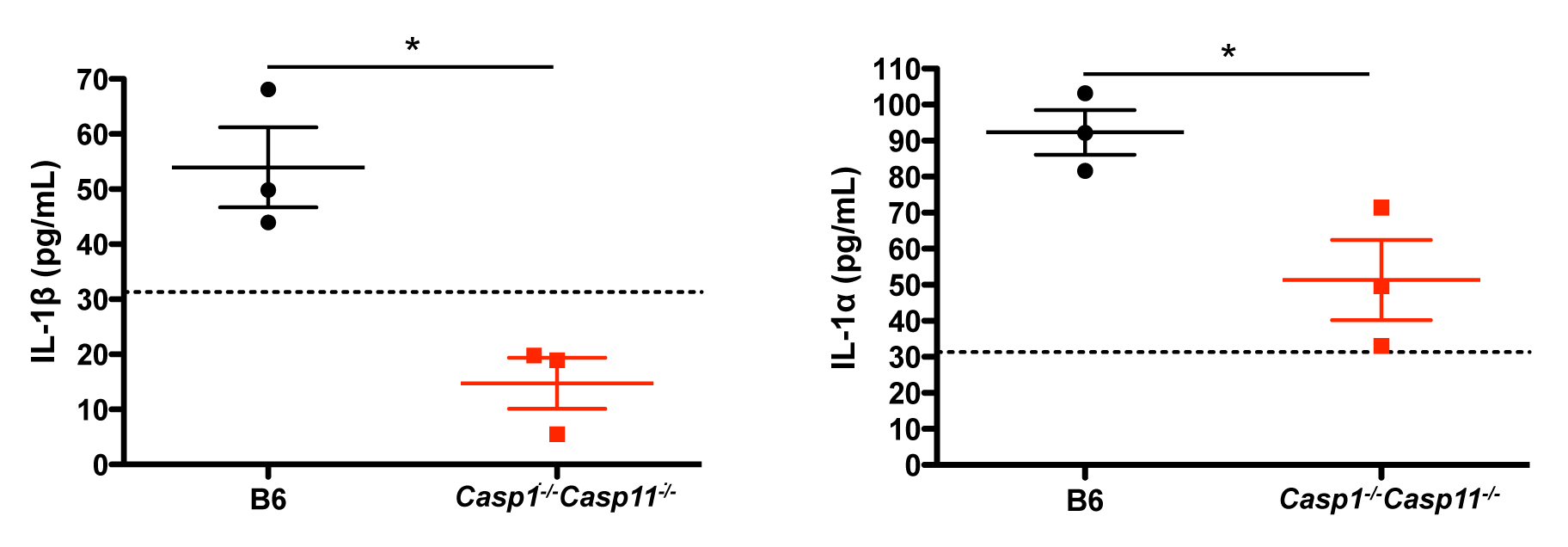

Supplement: Figure S5 — Both IL-1α and IL-1β secretion are caspase-1/caspase-11-dependent in vivo . 8–12 week old B6 or Casp1−/−Casp11−/− mice were infected intranasally with 1×10^6^ ΔflaA Lp. BALF was collected 24 hours post-infection, and levels of IL-1α and IL-1β were measured by ELISA. Graphs show the mean ± SEM of three mice per group. Dashed line represents the limit of detection. * is p<0.05 by unpaired t-test. (TIF) [file ppat.1003400.s005.tif]

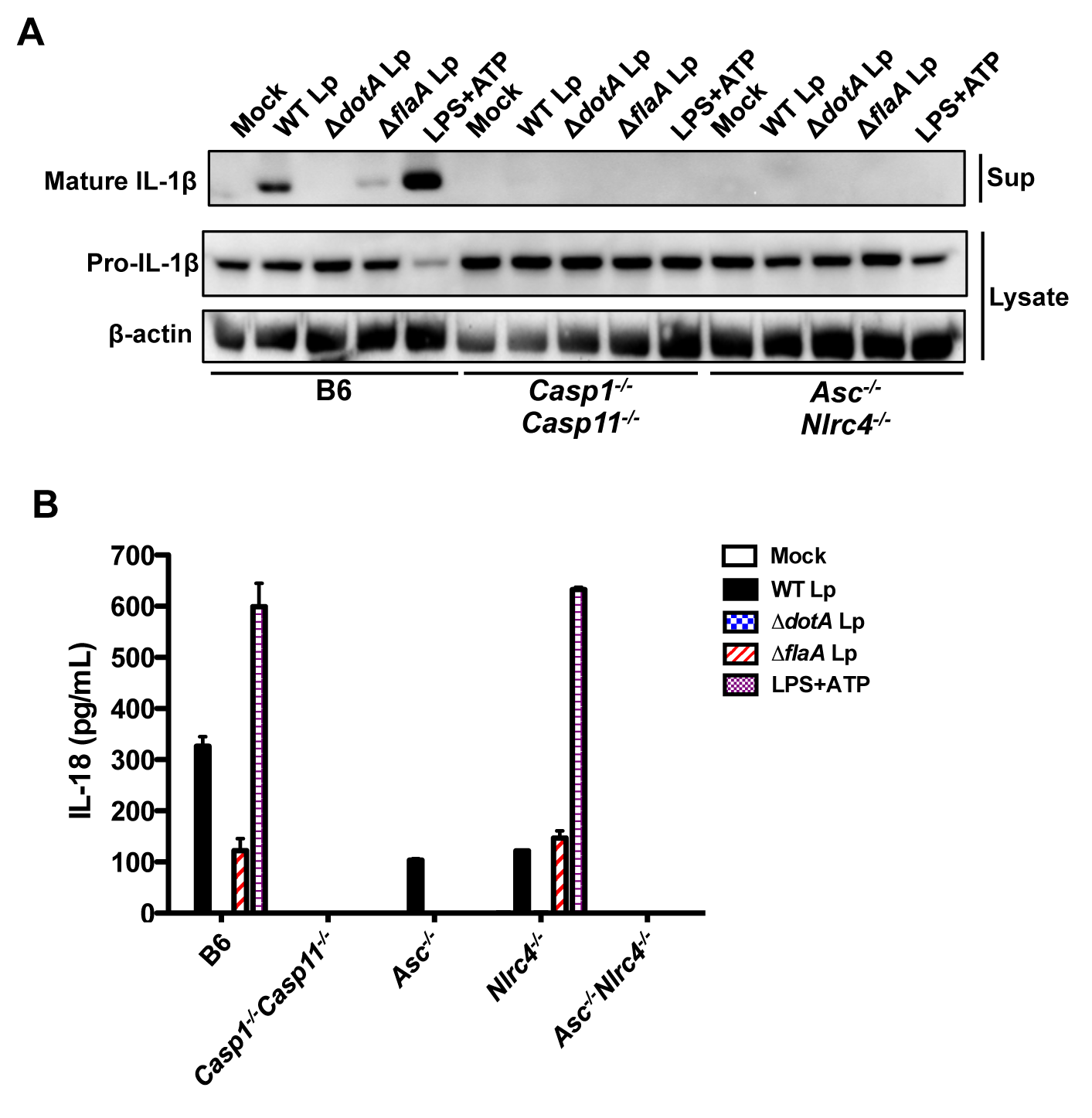

Supplement: Figure S6 — Mature IL-1β secretion is not always concordant with cell death. B6, Casp1−/−Casp11−/−, Asc −/−, Nlrc4 −/−, or Asc−/−Nlrc4−/− BMDMs were primed with 0.5 µg/mL LPS for 2.5 hours and infected with WT L. pneumophila (Lp), ΔdotA Lp, ΔflaA Lp or PBS (mock infection) for 4 hours or treated with 2.5 mm ATP for 1 hour. (A) Levels of mature IL-1β in the supernatants, and full-length IL-1β (pro-IL-1β) and β-actin (loading control) in the cell lysates were determined by immunoblot analysis. Data are representative of two independent experiments. (B) The level of IL-18 in the supernatants was measured by ELISA. Graphs show the mean ± SEM of triplicate wells. (TIF) [file ppat.1003400.s006.tif]

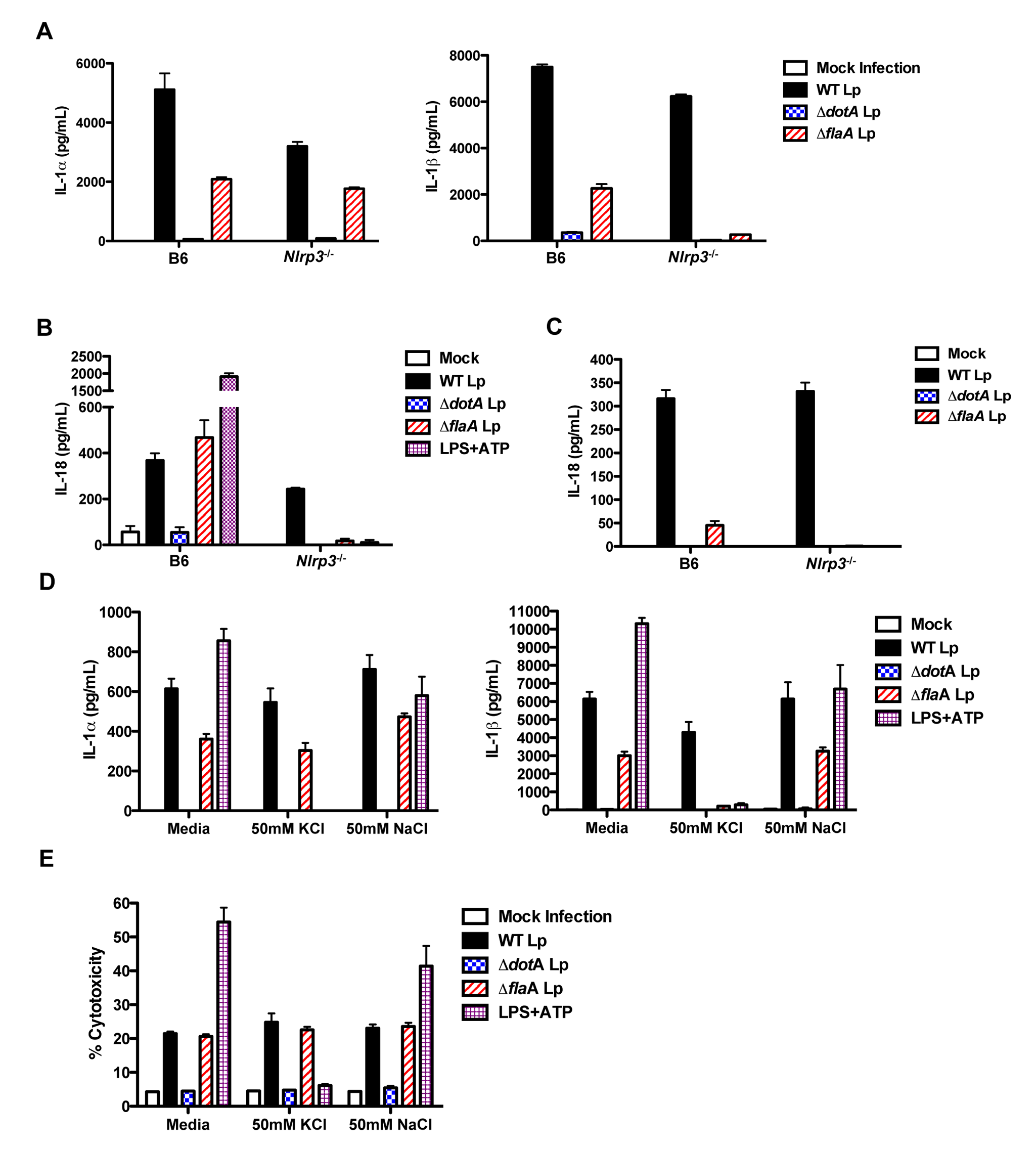

Supplement: Figure S7 — Flagellin-independent, NLRP3-dependent IL-1β secretion occurs independently of macrophage priming. (A) Unprimed B6 or Nlrp3−/− BMDMs were infected with WT L. pneumophila (Lp), ΔdotA Lp, ΔflaA Lp, or PBS (mock infection) for 16 hours. Levels of IL-1α and IL-1β in the supernatants were measured by ELISA. (B) B6 or Nlrp3−/− BMDMs were primed with 0.5 µg/mL LPS for 2.5 hours and infected with WT Lp, ΔdotA Lp, ΔflaA Lp or PBS (mock infection) or treated with 2.5 mM ATP for 4 hours. The level of IL-18 in the supernatants was measured by ELISA. (C) B6 or Nlrp3−/− BMDMs were infected with WT Lp, ΔdotA Lp, ΔflaA Lp, or PBS (mock infection) for 16 hours. The level of IL-18 in the supernatants was measured by ELISA. (D and E) B6 BMDMs were primed with 0.5 µg/mL LPS for 2.5 hours and infected with WT Lp, ΔdotA Lp, ΔflaA Lp or PBS (mock infection) or treated with 2.5 mM ATP for 4 hours. Where indicated, media alone, 50 mM KCl, or 50 mM NaCl were added prior to infection. (D) Levels of IL-1α and IL-1β in the supernatants were measured by ELISA. (E) Cell death was measured by LDH release. Graphs show the mean ± SEM of triplicate wells. Data are representative of two independent experiments (A–C). (TIF) [file ppat.1003400.s007.tif]

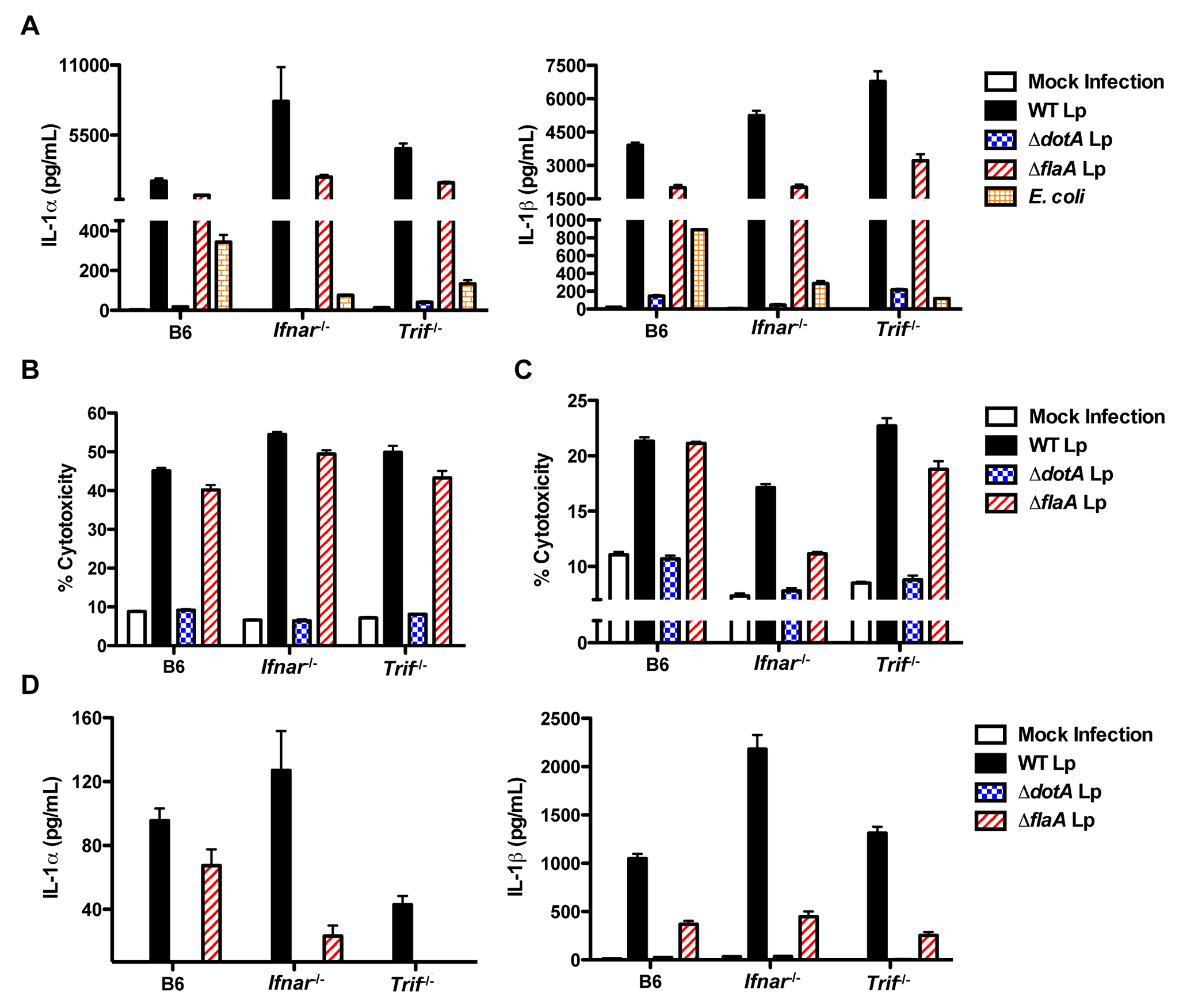

Supplement: Figure S8 — TRIF/IFNAR-independent IL-1 release occurs with Pam3CSK4-primed macrophages. (A) B6, Ifnar−/−, or Trif−/− BMDMs were primed with 0.4 µg/mL Pam3CSK4 for 4 hours and infected with WT L. pneumophila (Lp), ΔdotA Lp, ΔflaA Lp, E. coli, or PBS (mock infection) for 16 hours. The levels of IL-1α and IL-1β in the supernatants were measured by ELISA. (B) B6, Ifnar−/−, or Trif−/− BMDMs were primed with 0.4 µg/mL Pam3CSK4 for 4 hours and infected with WT Lp, ΔdotA Lp, ΔflaA Lp, or PBS for 16 hours. Cell death (% cytotoxicity) was measured by LDH release. (C) B6, Ifnar−/−, or Trif−/− BMDMs were primed with 0.5 µg/mL LPS for 2.5 hours and infected with WT Lp, ΔdotA Lp, ΔflaA Lp, or PBS for 4 hours. Cell death was measured by LDH release. (D) B6, Ifnar−/−, or Trif−/− BMDMs were primed with 0.5 µg/mL LPS for 2.5 hours and infected with WT Lp, ΔdotA Lp, ΔflaA Lp, or PBS for 4 hours. Levels of IL-1α and IL-1β in the supernatants were measured by ELISA. Graphs show the mean ± SEM of triplicate wells. Data are representative of two independent experiments. (TIF) [file ppat.1003400.s008.tif]

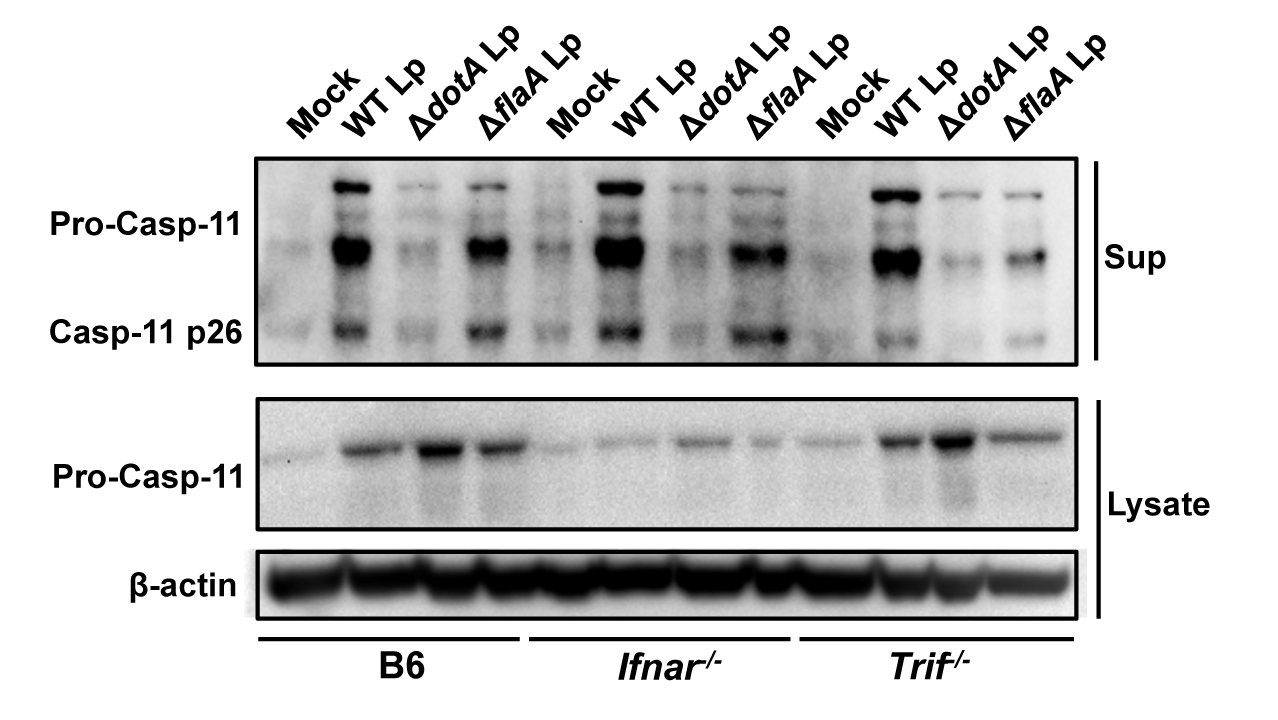

Supplement: Figure S9 — Caspase-11 is upregulated and secreted in an IFNAR- and TRIF-independent manner. Unprimed B6, Ifnar−/−, or Trif−/− BMDMs were infected with WT Lp, ΔdotA Lp, ΔflaA Lp, or PBS for 16 hours. Levels of full-length caspase-11 (pro-casp-11) and active caspase-11 (casp11 p26) in the supernatants, and pro-casp-11 and β-actin (loading control) in the cell lysates were determined by immunoblot analysis. (TIF) [file ppat.1003400.s009.tif]

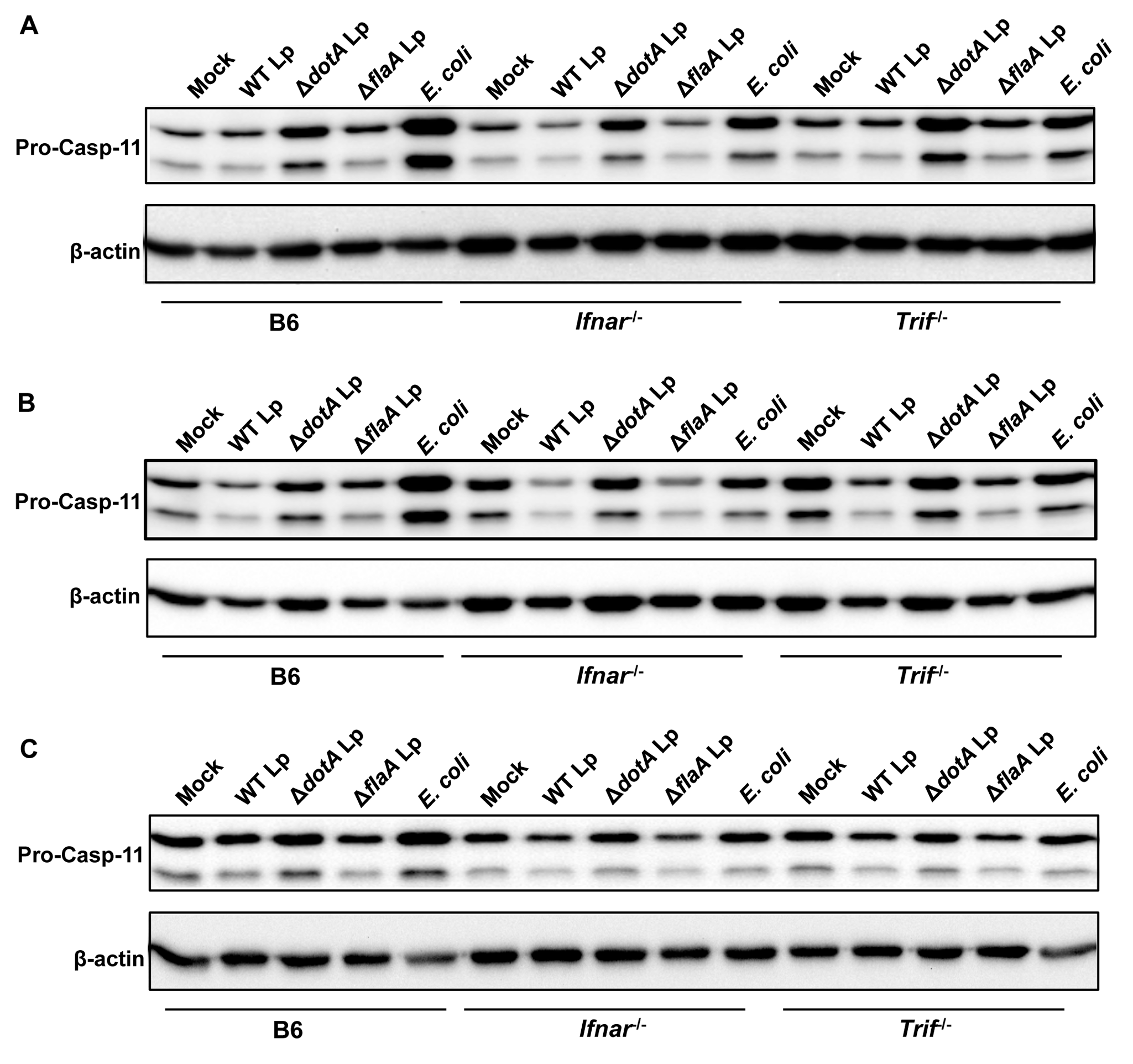

Supplement: Figure S10 — Detection of caspase-11 protein upregulation in cell lysates is moderate in response to L. pneumophila . (A) Unprimed B6, Ifnar−/−, or Trif−/− BMDMs were infected with WT Lp, ΔdotA Lp, ΔflaA Lp, E. coli, or PBS (mock infection) for 16 hours. (B) B6, Ifnar−/−, or Trif−/− BMDMs were primed with 0.4 µg/mL Pam3CSK4 for 4 hours and infected with WT Lp, ΔdotA Lp, ΔflaA Lp, E. coli, or PBS for 16 hours. (C) B6, Ifnar−/−, or Trif−/− BMDMs were primed with 0.5 µg/mL LPS for 2.5 hours and infected with WT Lp, ΔdotA Lp, ΔflaA Lp, E. coli, or PBS for 4 hours. Levels of full-length caspase-11 (pro-casp-11) and β-actin (loading control) in the cell lysates were determined by immunoblot analysis. (TIF) [file ppat.1003400.s010.tif]

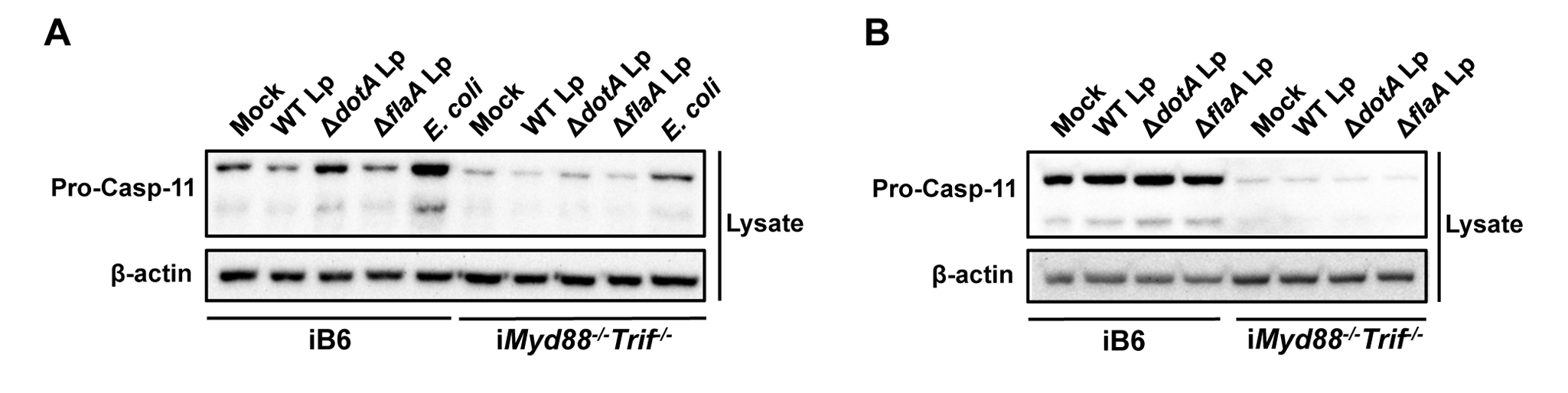

Supplement: Figure S11 — Caspase-11 is not upregulated in the absence of both MyD88 and Trif. (A) Immortalized B6 (iB6) or MyD88/Trif-deficient (iMyd88−/−Trif−/−) BMDMs were primed with 0.4 µg/mL Pam3CSK4 for 4 hours and infected with WT Lp, ΔdotA Lp, ΔflaA Lp, E. coli, or PBS (mock infection) for 16 hours. (B) iB6 or iMyd88−/−Trif−/− macrophages were primed with 0.5 µg/mL LPS for 4 hours and infected with WT Lp, ΔdotA Lp, ΔflaA Lp, or PBS (mock infection) for 4 hours. Levels of full-length caspase-11 (pro-casp-11) and β-actin (loading control) were determined by immunoblot analysis. (TIF) [file ppat.1003400.s011.tif]

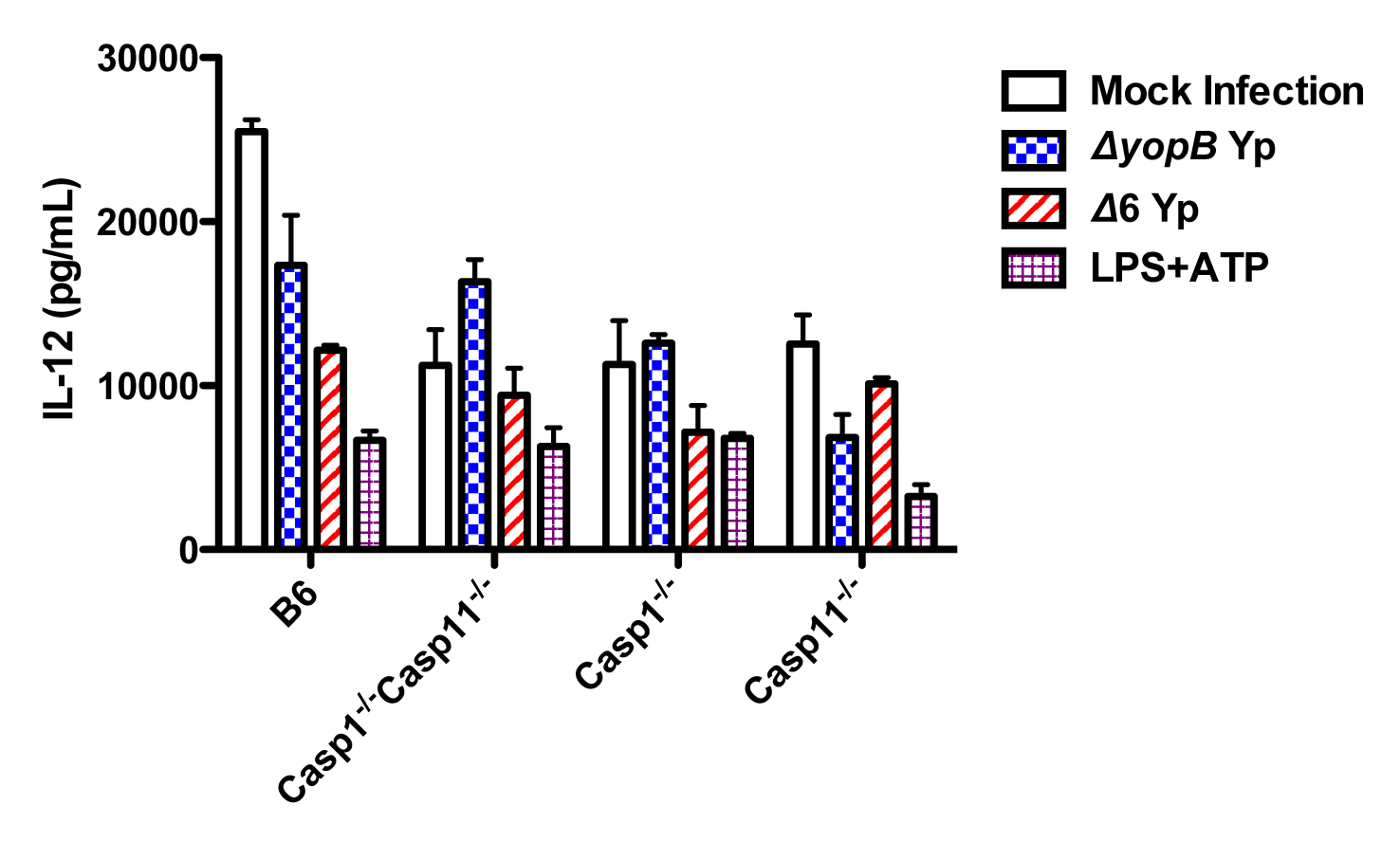

Supplement: Figure S12 — Caspase-11-deficient cells secrete comparable amounts of IL-12 in response to Y. pseudotuberculosis . B6, Casp1−/−Casp11−/−, Casp1−/−, or Casp11−/− mice were primed with 0.05 µg/mL LPS for 2.5 hours and infected with type III secretion system-deficient Y. pseudotuberculosis (ΔyopB Yp), effectorless Y. pseudotuberculosis ΔHOJMEK (Δ6 Yp), or PBS (mock infection) or treated with 2.5 mm ATP for 4 hours. The level of IL-12 p40 in the supernatants was measured by ELISA. Graphs show the mean ± SEM of triplicate wells. Data are representative of two independent experiments. (TIF) [file ppat.1003400.s012.tif]

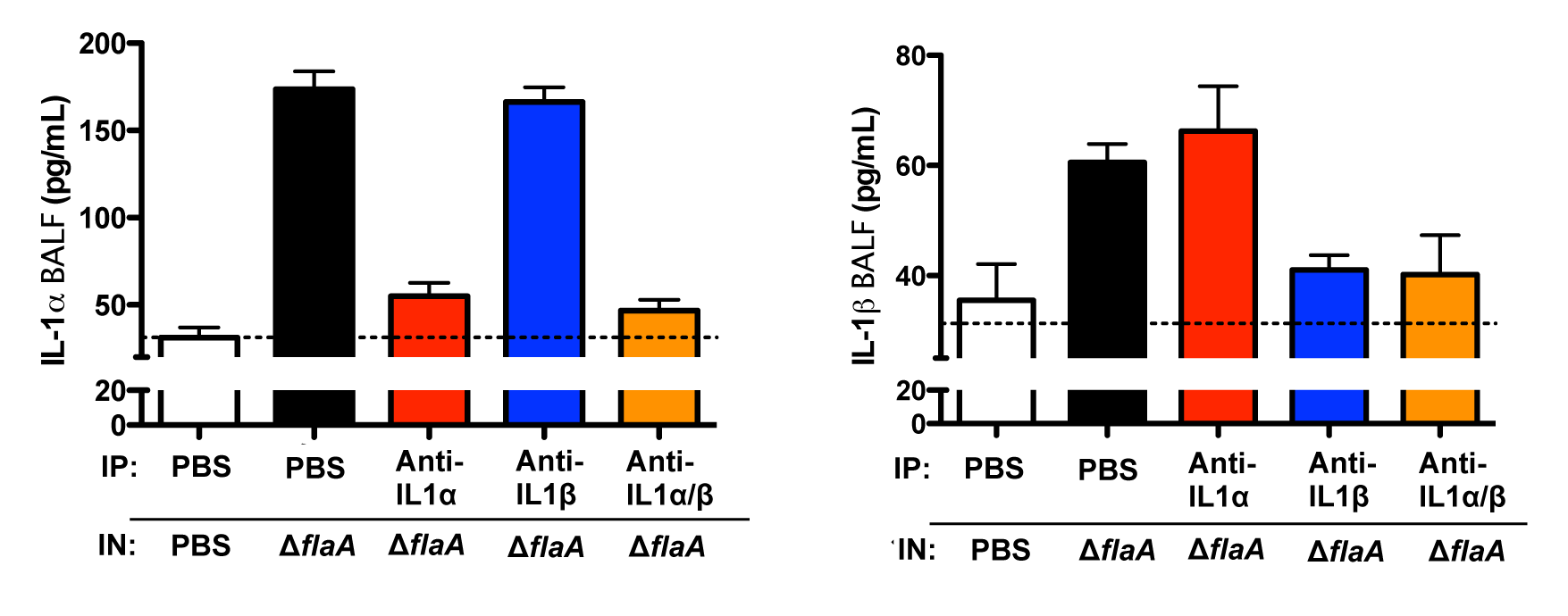

Supplement: Figure S13 — Intraperitoneally injected antibodies neutralize cytokine in the BALF. 8–12 week old B6 mice were injected intraperitoneally (IP) with either PBS, 100 µg anti-IL-1α antibody, 100 µg anti-IL-1β antibody, or 100 µg each of anti-IL-1α and anti-IL-1β (anti-IL-1α/β) 16 hours before infection. The mice were then infected with either 1×10^6^ ΔflaA Lp or mock infected with PBS intranasally (IN). 24 hours post-infection, bronchoalveolar lavage fluid (BALF) was collected and the levels of IL-1α and IL-1β were measured by ELISA. Labels indicate what was received intraperitoneally (IP) and what was received intranasally (IN). Graphs show the mean ± SEM of 8 mice per group and represent the pooled results of two independent experiments. (TIF) [file ppat.1003400.s013.tif]
